# Variance Decomposition of the Continuous Assessment of Interpersonal Dynamics (CAID) system: Assessing sources of influence and reliability of observations of parent-teen interactions

**DOI:** 10.1371/journal.pone.0292304

**Published:** 2023-10-18

**Authors:** Nolan E. Ramer, Sydney E. Fox, Samuel N. Meisel, Nicole Kiss, Jamie L. Page, Christopher J. Hopwood, Craig R. Colder

**Affiliations:** 1 Department of Psychology, University at Buffalo, SUNY, Buffalo, NY, United States of America; 2 Department of Psychiatry and Behavioral Sciences, Medical University of South Carolina, Charleston, SC, United States of America; 3 Department of Psychology, University of California Davis, Davis, CA, United States of America; 4 Center for Alcohol and Addiction Studies, Brown University, Providence, RI, United States of America; 5 Department of Psychology, University of Zurich, Zürich, Switzerland; University of Connecticut, UNITED STATES

## Abstract

The Continuous Assessment of Interpersonal Dynamics (CAID) is an observational tool that measures warmth and dominance dynamics in real time and is sensitive to individual, dyadic, and contextual influences. Parent-adolescent interpersonal dynamics, which conceptually map onto parenting styles, are an integral part of positive adolescent adjustment and protect against risky outcomes. The current study’s goal was to test the degree to which sources of influence on CAID data observed in a previous study of married couples generalize to a sample of parent-adolescent dyads. We examined data from ten raters who rated moment-to-moment warmth and dominance using CAID in a sample of 61 parent-adolescent dyads (*N* = 122) who were largely non-Hispanic White (62%) or African American (30%) based on parent report (adolescent *M* age = 14; 57% female). Dyads interacted in four different discussion segments (situations). We applied Generalizability Theory to delineate several sources of variance in CAID parameters and estimated within and between-person reliability. Results revealed a number of different influences, including the person, kinsperson (adolescent versus parent), dyad, rater, situation, and interactions among these factors, on ratings of parent-adolescent interpersonal behavior. These results largely replicate results from married couples, suggesting that the factors that influence ratings of interpersonal interactions largely generalize across sample types.

## Introduction

Theories of parenting, personality, and behavior change converge in positing that dynamic patterns of parent-adolescent interactions convey meaningful information regarding interpersonal functioning [1–4]. For example, dynamic systems theory contends that momentary parent-adolescent interactions shape long-term adolescent adjustment [2]. Although several prominent theoretical frameworks emphasize the importance of dynamic interaction patterns, most assessments of parent-adolescent interpersonal behavior provide a description of behavior that aggregates across long time-frames (e.g., weeks, months) and contexts [5, 6]. Such assessments do not provide insight into interpersonal processes that unfold during parent-adolescent interactions, such as the reciprocal nature of parent-adolescent interactions [7–9]. Tools that allow researchers to study parent-adolescent interpersonal dynamics as they unfold in real time are likely to provide important information about the nature of these dynamics and help us better understand their role in promoting healthy adjustment during adolescence. This, in turn, can help interventionists identify parent-adolescent dyads at risk for problematic interpersonal dynamics and new targets for behavioral change, such as identifying dyads who reciprocate warmth poorly and coaching parents to balance dominant behaviors, like limit setting, with warmth.

One observational tool that has shown promise in its ability to assess interpersonal dynamics is the Continuous Assessment of Interpersonal Dynamics system (CAID) [10–13]. The CAID system assesses interpersonal dynamics as they unfold in real time and models interpersonal processes at the individual and dyadic level, capturing contextual nuance and the reciprocal nature of these dynamics. The CAID has shown promise in understanding parent-child interactions, demonstrating that they can be reliably coded and linked to indicators of adjustment [12, 14, 15]. For example, our prior work with this sample examined whether interpersonal dynamics between parents and adolescents varied as a function of discussing various topics related to alcohol and cannabis use, and whether CAID parameters were associated with a variety of parenting and substance use outcomes [12].

However, the factors that might account for variation in parent-adolescent interpersonal dynamics as assessed by the CAID are not well understood. In a novel study that utilized a sample of married heterosexual couples, Fox et al. [16] applied Generalizability Theory to empirically investigate sources of influences on CAID parameters. Extending Generalizability Theory to understand potential sources of variation on ratings of interpersonal dynamics in adolescent-parent dyads is important because independence and autonomy are important developmental tasks of adolescence, and re-negotiating parent-adolescent interpersonal dynamics is a primary means to facilitate achieving healthy separation from parents [1, 17]. The purpose of the current study was to replicate and extend the findings from Fox et al. [16] in a sample of parent and adolescent dyads to better understand what might account for variability in parent-adolescent interpersonal processes across three different situations–a conflict discussion, alcohol use discussion, and cannabis use discussion—thereby enhancing the interpretation and generalizability of the most common CAID parameters across dyad types.

### Interpersonal assessment

**Fig 1:**
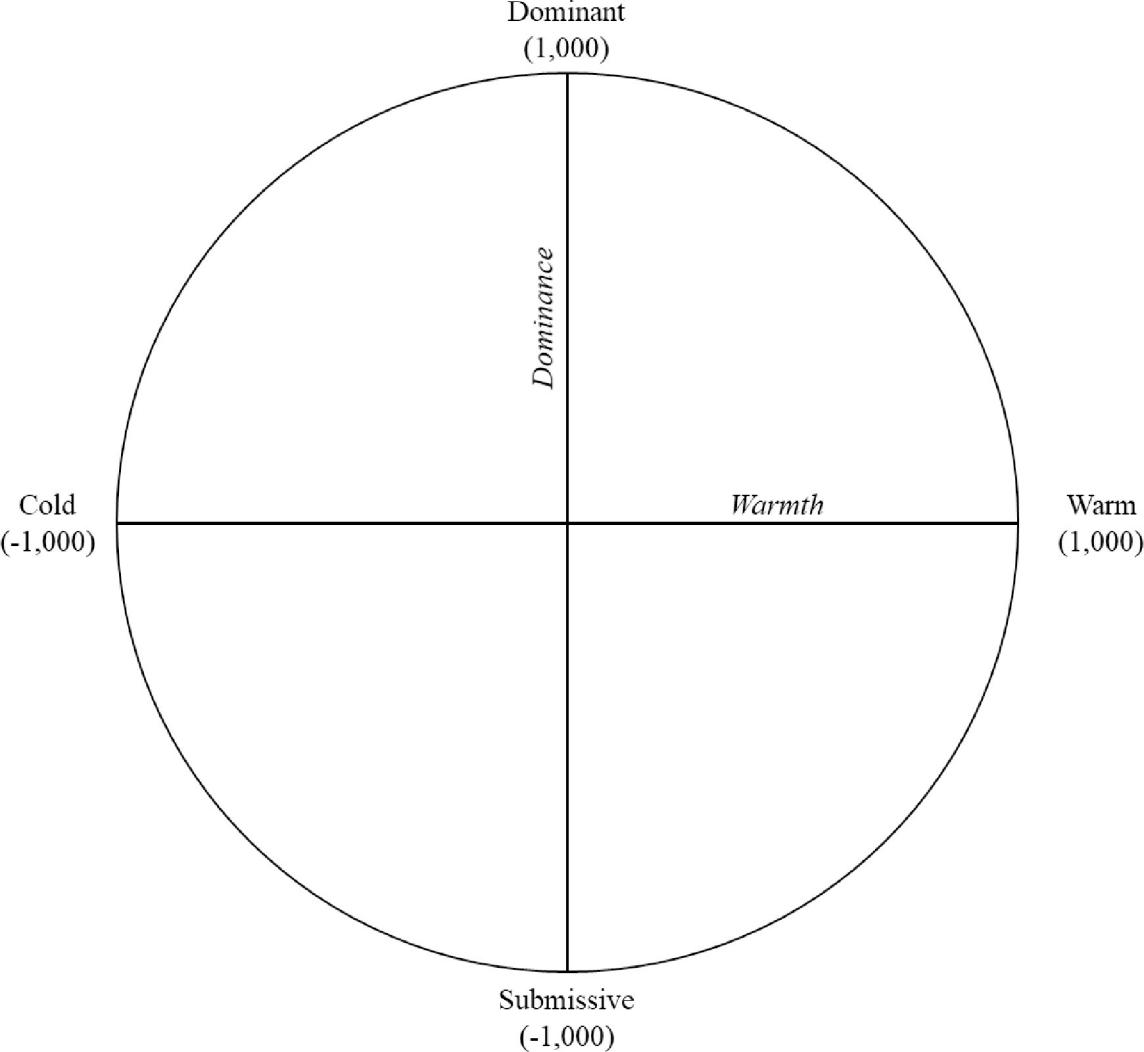
The interpersonal circumplex (IPC).

Interpersonal Theory posits that interpersonal interactions can be organized around a circumplex structured by the orthogonal dimensions of agency/dominance that captures variations from dominance to submission, and communion/warmth that captures variations from warmth to coldness (*see* Fig 1, The Interpersonal Circumplex) [4]. Variation around the interpersonal circumplex reflects the nature or quality of behavior characterized by different blends of agency and communion (e.g., cold vs. cold-submissive vs. submissive) and variation from the center to the edge of the circumplex reflects the intensity of a behavior [18]. A strength of the interpersonal circumplex is its ability to measure interpersonal functioning across multiple domains (e.g., interpersonal behaviors, problems, sensitivities), contexts (e.g., conflictual versus neutral topics), and time frames (e.g., seconds, days, years) [for a review see 19].

Several frameworks of parenting are similarly organized around the dimensions of warmth and dominance, and a core principle of these frameworks is that parent-child interpersonal behavior varies as a function of context [20–22]. A critique of current measures of parent-adolescent interpersonal functioning, as well as interpersonal behavior more broadly, is that they predominantly capture global assessments that are averaged over various contexts [23–26]. As such, they cannot assess how and to what extent parental responsiveness and control varies as a function of context and how adolescents respond to such parenting variability. For example, a parent may exhibit relatively high warmth and low dominance on average in a relationship with their adolescent. However, this same parent may be prone to sudden declines in warmth and increases in dominance during a stressful interaction with their adolescent, such as when responding to problem behavior or discussing contentious topics [27]. These dynamics could play an important role in predicting effectiveness of parenting as well as adjustment outcomes but may be missed through global assessments. Even commonly used observational assessments of parent-adolescent relationships predominantly rely on averages that collapse across discussions or moments within discussions [7, 8, 28–30]. The focus on aggregate measures of interpersonal behavior likely is a function of the availability of assessment tools, ease of administration, and reliability statistics for these measures compared to assessment measures of momentary interpersonal behavior.

Not only do measures that aggregate across time and/or context likely miss important features of interpersonal functioning, but they also do not align with theoretical formulations that emphasize the importance of moment-to-moment quality of interpersonal transactions [2, 9]. Measurement tools are needed to capture how relationship dynamics unfold across time and contexts [11]. Reliably measuring moment-to-moment behaviors will allow for tests of core tenets of parenting and interpersonal theory. For example, interpersonal theory provides detailed hypotheses regarding how the warm or dominant behaviors of one person invites the behavior of the other person (i.e., warmth and dominance complementarity). A parent high in dominance and low in warmth during a particular moment of an interaction likely elicits submission and coldness from their adolescent in that moment (high dominance but low warmth complementarity), which then could influence the quality of the discussion, how receptive adolescents are to parents’ messages, and be associated with important clinical outcomes, like adolescent substance use [8]. Further, deviations from complementarity have been hypothesized to be associated with psychopathology [31] and important therapeutic processes [32, 33]. For instance, Nilsen et al. [15] tested whether warmth and dominance complementarity varied between children and their mothers as a function of child Attention Deficit-Hyperactivity Disorder (ADHD) symptoms. They found lower warmth complementarity when children exhibited high ADHD symptoms, suggesting that parent-child dyads with a lower tendency to reciprocate warmth also had a higher likelihood of severe child ADHD symptoms. Assessing complementarity requires tools that can capture variation within interactions as complementarity is thought of as a transaction that occurs over the course of an interaction [13]. Accordingly, the current study focuses on the Continuous Assessment of Interpersonal Dynamics (CAID), an instrument designed to capture variations in interpersonal behavior over the course of interactions.

### Continuous assessment of interpersonal dynamics

The CAID is an observational coding method where raters use a computer joystick to continuously assess changes in warmth and dominance during an interaction [13]. The CAID has successfully been applied to studies of married couples and romantic partners [10, 11, 16], patients and therapists [32, 34], unacquainted undergraduates [13, 35], and parents and their children [11, 12, 14, 15]. These studies have yielded novel insights into interpersonal dynamics that would be missed with typical, aggregate assessments of interpersonal behavior. For example, Hopwood and colleagues [11] found that warmth and dominance complementarity vary as a function of dyad familiarity as well as the topic of the conversation. Specifically, warmth complementarity was higher among unfamiliar dyads and dominance complementarity was stronger among familiar dyads. They also found that warmth complementarity was lowest while dominance complementarity was highest during conflict tasks. These findings underscore the dynamic nature of interpersonal behavior across people and situations, highlighting the importance of continuous assessment of interpersonal behavior as it progresses in real time.

#### CAID parameters of interest

CAID offers a number of useful parameters to assess interpersonal process (*see* Table 1) [16], including individual level parameters of time series data (i.e., between and within person mean and variability estimates) and parameters that reflect associations between time series either within a person (shape) or across people (complementarity). Concerning individual level parameters, mean warmth and dominance CAID parameters represent aggregated scores of an individual across moments over the course of an interaction. These values indicate average warmth and dominance of a person in a given situation, and are analogous to interaction summary scores common in the parenting literature. Although similar to personality traits, the mean estimates can be aggregated within a situation or across situations and, therefore, must be interpreted in the context of a given situation or relationship.

**Table 1:** Observed indices of interpersonal behaviors.

Individual Behaviors	Computation	High Scores Indicate	Low Scores Indicate
*Average Warmth*	Mean of person’s warmth time-series	Warm; Connected	Cold; Distant
*Average Dominance*	Mean of a person’s dominance time-series	Assertive; Controlling	Passive; Submissive
*Warmth Variability*	Standard Deviation of person’s warmth time-series	Variability in warmth	Constancy in warmth
*Dominance Variability*	Standard Deviation of person’s dominance time-series	Variability in dominance	Constancy in dominance
*Warmth-Dominance Shape*	Correlation of person’s warmth and dominance time-series	Extraverted/Authoritative (warm-dominant) *and/or* Withdrawn/Uninvolved (cold-submissive)	Agreeable/Permissive (warm-submissive) *and/or* Antagonistic/Authoritarian (cold-dominant)
**Dyadic Behaviors**			
*Mean-Level* *Warmth Complementarity*	Absolute value of the discrepancy between individuals’ mean warmth	Low Complementarity	High Complementarity
*Moment-to-Moment* *Warmth Complementarity*	Cross correlation of individuals’ warmth time-series	High Complementarity	Low Complementarity
*Mean-Level* *Dominance Complementarity*	Absolute value of the discrepancy between individuals’ mean dominance	Low Complementarity	High Complementarity
*Moment-to-Moment Dominance Complementarity*	Correlation of individuals’ dominance time-series	Low Complementarity	High Complementarity

*Note*: We collected time-series using the Continuous Assessment of Interpersonal Dynamics (CAID; [13, 47]). Several precautions should be taken when correlating time-series data (e.g., Warner, 1998). We computed cross-correlation functions and examined correlations between relevant residual time-series data after accounting for any linear trends. Results did not substantively differ across these indices.

Variabilities can be computed around these means, which model the degree to which individuals vary from their mean level across different moments of an interaction or across all situations. Again, this variation must be interpreted within the context of the situation or relationship. For example, a parent may exhibit high variability in warmth with their child during a mutual task, becoming warm in response to their child’s cooperation and cold in response to their child’s opposition, but may display little variability in dominance given their authoritative position over the child. To estimate parameters that reflect associations of time series within and across people, the data is detrended for time, allowing for interpretation of momentary fluctuations while controlling for time-related trends [13]. Shape refers to the within-person time series correlations between warmth and dominance. Shape can provide insight into whether an individual becomes more dominant as they become warmer [32, 36] and can be estimated within a specific situation or across situations.

Finally, CAID data permits estimating complementarity at both the mean-level and moment-to-moment level (*see* Table 1) [16]. Complementarity reflects a dyadic level association between individuals’ interpersonal behavior. Warmth complementarity occurs when warmth from one interacting partner is reciprocated with warmth by the other. Dominance complementarity represents an inverse relationship, such that increased dominance from one individual elicits increased submissiveness from the other, and vice versa. Mean-level complementarity reflects average similarity between aggregated warmth or dominance scores within a situation or across all situations. Moment-to-moment complementarity reflects the correlation between time series across dyadic partners as a specific situation unfolds or across all situations as they unfold. Research suggests that mean and moment-to-moment level complementarity capture different processes [13]. For example, a parent-adolescent dyad may have high warmth complementarity on average, but certain interactions, such as discussing rule breaking, may elicit low warmth complementarity as the discussion unfolds. In order for these parameters to be useful for interpretation, however, these parameters must be assessed reliably and researchers must understand what factors influence them. Generalizability Theory offers tools to accomplish these goals.

### Generalizability Theory

#### G and D study

Generalizability Theory (GT) [37] is an extension from classical test theory which allows for modeling sources of influence that contribute to the variability of an observed measure. GT involves two consecutive sets of analyses: the “G study” and “D study”. The G (generalizability) study allows for isolating and estimating variation in potential sources of influences on CAID parameters [37, 38]. This is crucial for the interpretation of CAID parameters, as it allows researchers to parse apart whether observed scores vary as a function of individual, dyad, or measurement level processes. Estimating sources of influence on CAID scores is important because it facilitates accurate interpretation of parameters [16]. For example, differences in mean-levels of dominance between a parent and adolescent may reflect a stable quality of the parent-adolescent relationship. Alternatively, discussing certain topics can shift otherwise stable patterns of interaction (e.g., substance use, sexual activity, house rules) [39]. The current study focuses on estimating sources of variance on interpersonal behavior including the person, kinsperson (the role of parent versus adolescent), dyad, rater, situation, and two-way interactions between these potential influences.

Using the parameter estimates from the G study, reliability estimates for CAID-based inferences are computed in the D (decision) study [38, 40, 41]. Features about the CAID system, such as the number of situations needed to make accurate inferences about interpersonal behavior or whether variation across situations is due to important situational features or the progression of interpersonal dynamics, can be determined through G study estimates. The G and D study together enable identification of sources of variance on CAID parameters of warmth and dominance, disaggregation from measurement error, and evaluation of CAID results based on the extent of influence each source has on these ratings.

#### Summary of prior study

In their study of married heterosexual couples using the CAID system, Fox et al., [16] found support for the application of Generalizability Theory to interpret CAID data (see Tables 3–5 for comparison of results between their study and the current one). They found that a number of sources significantly predicted variance across individual behavioral indices and dyadic complementarity (G study). Specifically, with regard to indices of individual behavior, they found that the couple had the largest influence on individual mean warmth followed by the couple by situation interaction, person, situation, and couple by rater interaction. The person was the largest source of variance on individual mean dominance followed by the person by situation interaction. Robust rater and couple (and their interactions) effects were observed for predicting individual variability in warmth and dominance. Specifically, the rater was the largest source of variance on individual warmth variability, followed by the couple, couple by situation interaction, and couple by rater interaction. Like warmth variability, the rater was the largest source of variance on individual dominance variability followed by the couple by situation and couple by rater interactions. Notably, the person by situation interaction was the only significant source of variance predicting individual shape but accounted for nearly 25% of the variance in shape. With regard to indices of dyadic complementarity, the couple and couple by situation interaction were large and similar magnitude sources of variance on dyadic mean warmth complementarity. The rater and couple by situation interaction were both large sources of variance on dyadic mean dominance complementarity followed by a smaller couple effect. Lastly, the couple by situation interaction was the only significant influence on moment-to-moment warmth complementarity. The couple was the largest source of variance on moment-to-moment dominance complementarity followed by the couple by situation interaction and rater.

With regard to individual and dyadic reliability of estimates (D study), Fox and colleagues [16] found that reliabilities ranged from poor to excellent. Specifically, with regard to individual-level estimates, average warmth and dominance estimates generally exhibited good to excellent reliability within individual situations, averaged across all situations, within individuals, and between different situations (with the exception of warmth). In contrast, warmth and dominance variability estimates exhibited poor to fair reliability across situations with the exception of good reliability when averaged across all situations. Shape reliabilities were poor within single situations and across different situations, but were good when averaged across all situations and within individuals. With regard to dyadic-level complementarity estimates, reliability averaged across all situations and within individuals were typically good or excellent for mean-level and moment-to-moment warmth and dominance complementarity estimates. Estimate reliabilities were much more variable when examined within a single situation or examined across different situations.

In summary, Fox et al., [16] found that a number of sources accounted for significant variance in warmth and dominance estimated at the individual and dyadic level. The couple and rater as well as the person by situation and couple by situation tended to be significant sources of variance across many estimates. The couple by situation interaction accounted for a substantial portion of all dyadic-level complementarity estimates. They also found that reliable estimates could be found within individual situations, but reliability improved when more data across more situations were included in estimates.

### Current study

In order for the CAID to provide novel insights into parent-adolescent interpersonal dynamics as they unfold in real-time, information is needed pertaining to the sources of influence on CAID parameters (G study) and the reliability of the CAID (D study). The aim of the current study is to apply GT to CAID data in a sample of parent-adolescent dyads to test the degree to which the results from Fox et al. [16] in a sample of heterosexual couples generalize to interactions between parents and adolescents. We did not preregister hypotheses, but generally expected the findings from the Fox et al. [16] study to replicate.

## Methods

The University at Buffalo Institutional Review Board (IRB) approved this study (STUDY00003198). Written consent and assent were obtained from caregivers and adolescents.

### Participants

Parent and adolescent dyads were recruited through letters and flyers sent to families that had previously participated in studies in our laboratory and indicated a desire to be contacted about future studies. The sample included 61 parent-adolescent dyads (*N* = 122), which is consistent with most CAID studies that examined intensive observations of micro-level processes [11, 13, 15, 32, 34, 42–44], with a few exceptions [10, 14, 16, 35]. Average age of adolescents was 14.02 (*SD* = 1.53), 57.38% were female, and a majority of parents were White (N = 38, 62.30%) or Black or African American (N = 18, 29.51%). The average age of parents was 46.40 (*SD* = 6.51), and all but one parent was female. Most parents were married (N = 31, 50.82%) or divorced (N = 16, 26.23%), followed by single (N = 12, 19.67%) or living with a romantic partner (N = 2, 3.28%). Many of the parents had completed college (N = 22, 36.07%) or graduate school (N = 19, 31.15%), and the average household income ranged from $61,000–70,000, with a small percentage of families receiving public assistance income (N = 3, 4.92%).

### Procedures

All assessments were conducted in the university’s research lab. After obtaining consent and assent from parents and adolescents, respectively, research assistants (RAs) separated the dyads into different rooms to complete initial assessments. The Issues Checklist [45, 46] was completed by both adolescent and parent and was used to assess issues that often elicit conflict and the feelings evoked when discussing them using a 5-point scale (1 = calm to 5 = angry). The RA scored responses to identify the top eight issues that both participants reported and that generated anger. These top issues were selected from pre-printed and laminated issue cards and divided into 2 piles of 8 cards for each participant in the dyad. The adolescent pile was shuffled, and the parent’s pile was reordered to match the randomized order of the adolescent pile. All of this was done while participants completed the other self-report assessments on parenting and adolescent behavior, which are not of interest in the current study.

Dyads were then reunited in a private room set up to with two comfortable wingback chairs angled to face each other to complete discussion tasks. Participants were instructed to remain seated in the designated chair to ensure proper audio-visual data collection. Following a four-minute warm-up task that consisted of a discussion about the things their family likes to do together, the dyad completed three nine-minute discussions (alcohol, cannabis, and conflict issues) in a randomized order. For all discussions, the participants were told that it was not important to get through all discussion prompts, that they should not feel rushed, and to move on to another prompt once they have said all they had to say about a given topic. During the alcohol and cannabis discussions, RAs provided participants with a list of alcohol and cannabis-related prompts and instructed them to discuss their family’s beliefs about them. The complete list of issues can be found in S1 Appendix–Alcohol and Cannabis Discussions. The conflict discussion topics were selected by the RA based on areas of disagreement reported earlier. Dyads were asked to discuss the issues and come to an agreement about them before moving on to the next topic. Issue cards were then given to participants at the beginning of the observational session. Sessions generally ranged from 1 ½ to 2 hours in length.

### Measures

#### CAID

All CAID coding procedures and training materials were adapted from training materials and scripts originally developed by Pamela Sadler and Erik Woody [13] (see S2 Appendix–Joystick Manual for additional details on coding training adapted from [47, 48]). Interpersonal dynamics were assessed for adolescents and parents at half-second intervals throughout the four discussion types (warm-up, cannabis, alcohol, and conflict issues). Consistent with dyadic coding procedures outlined by Sadler et al. [13], undergraduate research assistants (five men and five women) watched video recordings of the interactions and continuously scored warmth and dominance by moving a computer cursor on a cartesian plane using a computer joystick and continuous measurement software (DARMA) [49]. The cartesian plane was defined by dominance on the y-axis and warmth on the x-axis. Locating the cursor within these coordinates allowed for variable levels of warmth and dominance to be rated simultaneously (see Fig 1). Before coding, research assistants completed 8 hours of training on the CAID system in order to establish standardized parameters for verbal and non-verbal displays of warmth and dominance. Then, upon meeting adequate inter-rater reliability on practice codes (.40 or greater), five research assistants coded each dyad. Data from the coder with the lowest overall comparative reliability for each dyad was omitted from analyses. Data from the other four coders were retained to assess inter-rater reliability in an effort to control for systematic biases which might influence a coders’ interpretation of interpersonal processes. All four interactions together included 31 minutes of interaction, had two data points per second, and included ratings from four coders per situation, resulting in 14,880 CAID data points used in analyses. While the sample size was small (61 dyads), this is consistent with prior work [13] and the intensive nature of the data from 122 participants yields a large number of data points that typically provides adequate power for Generalizability Theory Analysis [35].

### Data analytic plan

Descriptive and correlational statistics for CAID parameters were calculated first. To compute average warmth or dominance, the mean of all parents’ and teens’ warmth or dominance within each situation and overall was calculated. For warmth and dominance variability, we calculated all parents’ and teens’ standard deviations within each situation and averaged across all four situations (i.e., overall). Shape was calculated as each individual’s correlation between their warmth and dominance time-residualized time series data across all situations and for each situation. Time series were residualized to avoid confounding moment-to-moment dynamics with linear trends.

#### Estimating complementarity

Following Sadler and colleagues’ [13] procedures, four types of complementarity were estimated: mean-level and moment-to-moment complementarity for warmth and dominance. Dominance complementarity is when one individual is dominant while the other is submissive in a given moment. Warmth complementarity occurs when both individuals in the dyad are exhibiting similar levels of warmth. To calculate dominance complementarity at the mean-level, one individual’s dominance scores was averaged across the entire interaction. Each individual’s average dominance score was then added together within a dyad. The absolute value of this sum denotes the dyad’s mean-level dominance complementarity. Because high dominance (dominant) behaviors are scored as positive and low dominance (submissive) behaviors are coded as negative, values closer to zero indicate higher dominance complementarity (see Fig 1). For example, if the parent’s average score was 300 and the teen’s average score was -300, they would yield a complementarity score of 0, suggesting maximum mean-level dominance complementarity because the parent tends to be dominant on average while the teen tends to be submissive on average. If the teen’s average score were 200, this would yield a complementarity of 500, suggesting relatively low mean-level dominance complementarity because both the parent and teen exhibit relatively high dominant behavior on average.

In contrast, high mean-level warmth complementarity is estimated by taking the absolute value of the difference between the parent and teen’s individual average warmth scores. Small absolute values indicate high warmth complementarity. For example, if a parent’s warmth score is 300 (warm) on average and the teen’s average warmth score is 300 (warm), their complementarity score would be 0 (see Fig 1). This would indicate maximum warmth complementarity because both the parent and teen tend to be similarly warm together on average. If the teen’s warmth score was -300 (cold), instead, this would yield a complementarity score of 600, suggesting low mean-level warmth complementarity because the parent is warm while the teen is relatively cold on average.

To calculate moment-to-moment estimates, we correlated the detrended time series between the two individuals. Again, detrending by regressing out time avoids confounding the moment-to-moment associations with linear trends. Since dominance complementarity means that scores are concurrently shifting in opposite directions, a correlation closer to -1 suggests that the dyad is achieving closer to maximum moment-to-moment dominance complementarity. In contrast, a larger positive correlation between the two individual’s detrended data would indicate more moment-to-moment warmth complementarity because both individuals shift in warmth in the same direction from one moment to the next.

#### Generalizability Theory analyses

Generalizability Theory analyses are typically conducted in two steps, as described above. In the G study, we examined the extent to which observed individual CAID variables varied as a function of a number of sources using Cranford and colleagues’ [50] analysis of variance model. This model allows for warmth and dominance scores of persons in a given situation at a given time to be analyzed into components of variance. Influences at the individual level included person, kinsperson (parent or adolescent), parent-adolescent dyad, situation, and rater. Sources of influence at the dyadic level included parent-adolescent dyad, situation, and rater. All relevant influences were treated as nested (e.g., kinsperson nested with dyads) and all possible interactions among non-nested influences (e.g., rater x dyad) were tested. G study models were estimated as linear mixed effect models, using restricted maximum likelihood (REML) estimation, in which all sources of influence on CAID parameters were modeled as random intercepts in order to increase generalizability of results [51, 52]. The observed score variance, σ^2^_x_ equals the sum of all identified variance components and their interactions:

$$\sigma^{2}{}_{x}=\sigma^{2}{}_{intercept}+\sigma^{2}{}_{person}+\sigma^{2}{}_{dyad}+\sigma^{2}{}_{situation}+\sigma^{2}{}_{rater}+\sigma^{2}{}_{kinsperson}+\sigma^{2}{}_{person*situation}+\sigma^{2}{}_{dyad*situation}+\sigma^{2}{}_{rater*situation}+\sigma^{2}{}_{person*rater}+\sigma^{2}{}_{dyad*rater}+\sigma^{2}{}_{kinsperson*situation}+\sigma^{2}{}_{kinsperson*rater}+\sigma^{2}{}_{error}$$ (1)

*Note*. For dyadic-level complementarity estimates, only the dyad, situation, rater, and their interactions were included. The intercept was modeled as a fixed and random effect and each source of influence and their interactions were modeled as random intercepts.

Percentage of total variance accounted for by each source of influence, estimated by dividing the individual variance component by the total observed variance, was presented because raw G study coefficients vary in size across models due to computational differences in parameter estimates.

In the D study, between-person and within-person reliability of change across situations were estimated [40, 41, 50]. We followed Cranford and colleagues’ [50] procedure of four D study coefficients, which are similar to inter-rater reliability coefficients such as intra-class correlations [also see 16, 51]. The first coefficient represents an estimate of the reliability of ratings within a single fixed situation (R_1F_) and informs questions like “how reliable is a CAID parameter estimate in one particular situation across all raters?”.


$$R_{1F}=\frac{\sigma^{2}{}_{PERSON}+(\sigma^{2}{}_{PERSON*RATER/4})}{\sigma^{2}{}_{PERSON}+(\sigma^{2}{}_{PERSON*RATER/4})+(\sigma^{2}{}_{ERROR/4})}$$ (2)

*Note*: This was calculated for each CAID parameter (see Table 5). To calculate this coefficient for a dyad, each σ^2^_PERSON_ was replaced with σ^2^_DYAD._

The second coefficient estimates the reliability of ratings aggregated across all raters and situations (R_KF_) and answers questions like “how reliable would a CAID result be for a parameter averaged across all of the situations across all raters?”.


$$R_{KF}=\frac{\sigma^{2}{}_{PERSON}+(\sigma^{2}{}_{PERSON*RATER/10})}{\sigma^{2}{}_{PERSON}+(\sigma^{2}{}_{PERSON*RATER/10})+(\sigma^{2}{}_{ERROR/4*10})}$$ (3)

*Note*: This was calculated for each CAID parameter (see Table 5). To calculate this coefficient for a dyad, each σ^2^_PERSON_ was replaced with σ^2^_DYAD._

The third coefficient estimates the extent to which behavioral observations obtained from one situation reliably relate to behaviors during different situations (R_1R_). This coefficient assesses the reliability of CAID parameters in a situation in which each person was measured in a situation and answers questions like “how reliable would a CAID parameter estimate be if it was derived from any of the situations (randomized across participants)?”. Both R_1F_ and R_KF_ estimates only take one situation into account, but the first reliability estimate (R_1F_) assesses generalizability to other similar situations (e.g., one conflict situation compared to another conflict situation), whereas this third reliability estimate (R_KF_) assesses generalizability from a single situation to other different situations (e.g., warm up activity compared to conflict). As such, R_1R_ will always be lower than R_1F_.


$$R_{1R}=\frac{\sigma^{2}{}_{PERSON}+(\sigma^{2}{}_{PERSON*RATER/4})}{\sigma^{2}{}_{PERSON}+(\sigma^{2}{}_{PERSON*RATER/4})+\sigma^{2}{}_{SITUATION}+\sigma^{2}{}_{SITUATION*RATER}+(\sigma^{2}{}_{ERROR/4})}$$ (4)

*Note*: This was calculated for each CAID parameter (see Table 5). To calculate this coefficient for a dyad, each σ^2^_PERSON_ was replaced with σ^2^_DYAD._

The fourth coefficient (R_C_) estimates the degree to which the within-person changes across situations are due to true changes in behaviors relative to measurement unreliability and answers questions like “how reliable are within-person changes for a CAID parameter estimate from one situation to the next?”.


$$R_{C}=\frac{\sigma^{2}{}_{PERSON*SITUATION}}{\sigma^{2}{}_{PERSON*SITUATION}+(\sigma^{2}{}_{ERROR/10})}$$ (5)

*Note*: This was calculated for each CAID parameter (see Table 5). To calculate this coefficient for a dyad, each σ^2^_PERSON_ was replaced with σ^2^_DYAD._

We modeled the number of raters as 10, the total number who contributed data, for all D study analyses except when estimating the reliability of raters within the same situation (R_1F_ & R_1R_), in which we modeled the number of raters as 4, the number who rated a given situation. Results from the G study were used to calculate reliability coefficients. As such, all CAID parameters were treated as random intercepts. We performed these analyses in R using the lme4 package [52] and the script and the raw data is publicly available on the Open Science Framework (OSF) repository (DOI 10.17605/OSF.IO/NKASU).

## Results and discussion

### Descriptive statistics

#### Mean levels and variability

Descriptive and correlational statistics are reported in Table 2 and are also reported in Meisel et al. [12]. On average, parents exhibited greater overall warmth and dominance than adolescents. For both parents and adolescents, warmth was highest during the warmup and lowest during the conflict discussion. Parents were most dominant during the conflict discussion but teens were most submissive when discussing substance use. Adolescents and parents exhibited significant variability in warmth and dominance throughout the interaction, although warmth variably was relatively low compared to dominance variability for both teens and parents.

**Table 2:** Means, (SDs), and Bivariate Correlations of Kinspersons’ Interpersonal behaviors across situations.

Individual Behaviors	Overall	Warm Up	Alcohol	Cannabis	Conflict
**Average Warmth**					
Parents’	115.11_(102.49)_	173.15_(76.78)_	110.10_(92.89)_	101.03_(103.06)_	79.36_(111.43)_
Teens’	84.71_(121.62)_	134.21_(94.51)_	72.45_(127.96)_	74.86_(115.74)_	60.16_(132.96)_
*Kinsperson Correlation*	.59***	.59***	.61***	.47***	.60***
**Average Dominance**					
Parents’	119.19_(83.31)_	72.47_(69.45)_	132.98_(75.68)_	126.01_(87.73)_	142.61 _(82.81)_
Teens’	-45.85_(94.74)_	-31.10_(80.57)_	-65.63_(93.67)_	-62.29_(102.94)_	-23.05_(94.48)_
*Kinsperson Correlation*	-.56***	-.28	-.71***	-.65***	-.61***
**Warmth Variability**					
Parents’	72.19_(26.42)_	65.25_(24.28)_	67.61_(18.70)_	67.43_(22.13)_	88.26_(32.38)_
Teens’	71.09_(27.38)_	64.73_(23.34)_	67.76_(26.83)_	67.08_(25.69)_	84.58_(29.20)_
*Kinsperson Correlation*	.54***	.50***	.50***	.43***	.54***
**Dominance Variability**					
Parents’	136.18_(34.75)_	118.20_(33.37)_	138.50_(32.72)_	145.75_(32.29)_	141.18_(35.02)_
Teens’	139.38_(32.45)_	122.29_(27.28)_	144.26_(32.55)_	145.36_(32.09)_	144.57_(32.33)_
*Kinsperson Correlation*	.72******	.67***	.72***	.72***	.69***
**Warmth-Dominance Shape**					
Parents’	-.03_(.26)_	.12_(.22)_	-.02_(.24)_	-.05_(.25)_	-.15_(.25)_
Teens’	.15_(.30)_	.25_(.27)_	.17_(.30)_	.19_(.29)_	.01_(.31)_
*Kinsperson Correlation*	.32***	.34	.20	.32*	.14
**Dyadic Behaviors**	**Overall**	**Warm Up**	**Alcohol**	**Cannabis**	**Conflict**
**Warmth Complementarity**					
Mean-Level	77.78_(73.13)_	66.12_(57.40)_	81.41_(72.93)_	81.45_(82.47)_	81.43_(77.12)_
Moment-to-Moment	.24_(.20)_	.25_(.21)_	.23_(.19)_	.22_(.20)_	.28_(.19)_
*Complementarity Correlation*	-.08	-.11	-.16	-.02	-.02
**Dominance Complementarity**					
Mean-Level	89.80_(66.32)_	74.43_(65.85)_	78.73_(52.40)_	81.17_(63.30)_	124.42_(71.48)_
Moment-to-Moment	-.56_(.23)_	-.45_(.24)_	-.62_(.18)_	-.62_(.22)_	-.56_(.23)_
*Complementarity Correlation*	.25*	.36**	.13	.25*	.35**

*Note*: Overall estimates were obtained by averaging ratings across all of the other situations. Standard deviations (SDs) were computed using the stats package (version 3.6.1) in R. SDs reflect the average variation in means across people within each situation and overall. Correlation coefficients indicate correlations between the variables in the two proceeding rows

(* p ≤ .05

** p ≤ .01

*** p ≤ .001).

#### Shape

One advantage of the CAID system is the ability to assess how warmth and dominance covary as situations unfold (i.e., shape). The within-person shape of warmth and dominance was near zero on average, consistent with prior work and theory that suggests that warmth and dominance are orthogonal across individuals [16, 18]. However, some correlations suggested some individuals’ warmth and dominance did meaningfully change together. For example, adolescents exhibited a weak, positive shape in the warm-up situation (*r* = .25), suggesting that, although the effect is small, adolescents became increasingly warm as their dominance increased during discussions of positive topics (e.g., planning a family vacation, discussing things that the family does for fun). Alternatively, parents had a weak, negative shape during the conflict situation (*r* = -.15), indicating that parents were more likely to become cold as they became dominant. We also found some evidence of a correlation between parent and adolescent shape when averaged across all situations (overall *r* = .32), as well as within individual situations.

This is similar to Fox et al.’s [16] results in which they found a weak, positive correlation between spousal shape when aggregated across all situations. This suggests that for parents whose warmth and dominance shifted in tandem, so did their adolescent’s and vice versa. Interestingly, the correlation of overall shape in our data was almost three times as large (*r* = .32) as Fox et al.’s [16] (*r* = .12). As with parents exhibiting more dominance, this may reflect the natural power structure in parent-adolescent relationships [1]. Parents may view increasing dominance and coldness as an effective means to control behavior or set limits, whereas elevated dominance and coldness in a married couple may reflect increased conflict. This finding may also reflect an emotional process in parents, as some evidence suggests that poor emotion regulation skills are positively associated with an authoritarian parenting style (i.e., low warmth and high dominance) [53]. Lastly, this larger shape effect could reflect genetic or socialization transmission of interpersonal processes across generations [14, 54].

#### Complementarity

Consistent with interpersonal theory and prior work [11, 12, 55], we found evidence of complementarity at both the mean and moment-to-moment levels. At the dyadic level, some dyads showed more similar levels of warmth or coldness, on average. The moment-to-moment warmth complementarity correlation suggested relatively weak complementarity across all situations (*r* = .24, SE = .20), suggesting weak changes in parent’s and adolescent’s warmth in response to one another. Other sources may be influencing moment-to-moment changes in warmth more strongly or the limited variability in warmth may be attenuating this correlation. Non-significant (*ps* > .05) correlations between mean-level and moment-to-moment warmth complementarity across and within each situation indicated that a dyad’s average tendency to be similarly warm was unrelated to fluctuations in warmth complementarity from one moment to the next. In other words, although the term complementarity is used to describe both of these patterns, they seem to capture independent processes.

With regard to mean-level dominance complementarity, parents tended to be more dominant than adolescents on average. Moment-to-moment dominance complementarity tended to be strong overall (*r* = -.56, SE = .23), indicating that as one person expressed more dominance, the other responded with submissiveness as discussions unfolded. Mean-level and moment-to-moment dominance complimentary correlations were significant but modest when averaged across all situations (*r* = .25, *p* < .05), during the warm up situation (*r* = .36, *p* < .01), the cannabis discussion (*r* = .25, *p* < .05), and the conflict situation (*r* = .35, *p* < .01), but they were not significantly correlated in the alcohol situation (*r* = .13, *p* < .05). These positive correlations suggest that dyads with high mean-level dominance complementarity tend to reciprocate dominance from one moment to the next. In other words, in contrast to warmth complementarity, mean-level and momentary complementary reflect related processes.

### G study results

We first assessed the relative influence of characteristics related to persons, kinsperson (adolescent versus parent), dyads, situations, raters, and their interactions on interpersonal behavior. Generalizability Theory was used to evaluate these influences and G study results are reported in Tables 3 and 4. No standard guidelines for determining effect sizes exist, so we reported and evaluated each source’s absolute and relative influence on each parameter [51] Each value is represented as a percentage of its influence on each parameter. Inference testing is computationally complex and subject to bias when estimates are close to zero. Therefore, we used Likelihood Ratio Tests (LRT) and absolute value size to determine significance of variance estimates [56]. We followed Fox et al.’s [16] procedures and interpreted values above 9%, the threshold at which p-values were consistently estimated, as significant and values above 25% as the most significant contributors on CAID parameters. All converted variance proportions are reported in Tables 3 and 4 and all variance parameters are reported in Table 6 in S1 Table.

**Table 3:** Partitioning of total variance in individual-level Kinspersons’ interpersonal behaviors (G study analyses).

	Indices of Individual Behavior–Current Study					Indices of Individual Behavior–Reproduced from Fox et al. (2021)				
	Mean		Variability		Shape	Mean		Variability		Shape
*Source of Variance*	*Warm*	*Dom*	*Warm*	*Dom*	*Warm-Dom*	*Warm*	*Dom*	*Warn*	*Dom*	*Warm-Dom*
σ^2^ Person	14.9%***	20.6%***	3.1%***	1.4%*	1.5%	10.6%**	42.8%***	4.8%*	2.0%*	6.4%*
σ^2^ Dyad	19.9%***	0.0%	5.0%*	6.8%***	4.0%*	25.1%***	0.0%	13.2%**	7.5%*	6.2%*
σ^2^ Situation	6.8%**	0.0%	5.3%***	3.2%**	6.5%*	9.6%**	1.7%	4.3%	7.8%*	6.5%*
σ^2^ Rater	10.0%***	0.0%	23.5%***	41.3%***	6.7%*	4.7%*	1.9%*	17.8%**	24.0%**	4.7%*
σ^2^ Person*Situation	3.6%***	8.2%***	2.3%***	0.4%	6.3%***	5.5%*	24.1%**	1.6%	3.1%*	28.8%***
σ^2^ Dyad*Situation	5.5%***	0.0%	5.2%***	5.6%***	3.1%*	12.1%**	0.0%	12.8%**	11.5%**	0.0%
σ^2^ Rater*Situation	2.2%***	0.2%***	3.6%***	1.0%***	2.7%***	3.8%*	1.8%*	6.1%*	9.6%**	1.6%*
σ^2^ Person*Rater	7.6%***	9.8%***	5.3%***	9.8%***	15.4%***	0.0%	7.1%*	0.0%	0.0%	3.3%
σ^2^ Dyad*Rater	14.3%***	0.0%	22.6%***	14.2%***	11.1%***	9.7%**	0.0%	10.7%**	11.0%**	0.0%
σ^2^ Kinsperson	2.2%	45.5%***	0.0%	0.0%	5.5%*	0.2%	0.0%	0.4%	0.0%	0.9%
σ^2^ Kinsperson*Situation	0.0%	2.8%***	0.0%	0.1%	0.4%	0.1%	0.6%	0.3%	0.0%	0.0%
σ^2^ Kinsperson*Rater	1.1%**	6.8%***	0.5%	3.1%***	2.3%***	0.0%	0.2%	0.2%	0.0%	0.0%
σ^2^ Residual Error	11.9%	6.2%	23.5%	13.3%	34.4%	18.6%	19.7%	27.9%	23.5%	41.6%

*Note*: Each column represents the respective CAID parameter, whereas each row represents the source of influence on CAID parameter variance. Possible sources of variance included persons (n = 122), dyads (n = 61), situations (n = 4), rater (n = 10), kinsperson (n = 2), and their relevant interactions. Variation proportions within each column sum to 100%, within rounding error. Significance for random effects were determined with Likelihood-Ratio tests within each parameter using lmerTest package in R

(* p ≤ .05

** p ≤ .01

*** p ≤ .001). Fox et al. [16] G study results are reported for ease of comparison, and person sex was assessed as a source of influence instead of kinsperson (adolescent versus parent).

**Table 4:** Partitioning of total variance in dyadic-level Kinspersons’ interpersonal behaviors (G study analyses).

	Indices of Dyadic Complementarity–Current Study				Indices of Dyadic Complementarity–Fox et al. (2021)			
	Mean-Level		Moment-to-Moment		Mean-Level		Moment-to-Moment	
*Source of Variance*	*Warm*	*Dom*	*Warm*	*Dom*	*Warm*	*Dom*	*Warm*	*Dom*
σ^2^ Dyad	30.2%***	30.2%***	3.6%	30.2%***	30.2%***	30.2%***	30.2%***	30.2%***
σ^2^ Situation	0.3%	30.2%***	0.2%	30.2%***	1.0%	1.3%	0.0%	30.2%***
σ^2^ Rater	30.2%***	30.2%***	30.2%***	30.2%***	30.2%***	30.2%***	5.2%**	30.2%***
σ^2^ Dyad*Situation	30.2%***	30.2%***	30.2%***	30.2%***	30.2%***	30.2%***	30.2%***	30.2%***
σ^2^ Rater*Situation	0.4%	30.2%***	0.0%	0.8%**	30.2%***	30.2%***	30.2%***	30.2%***
σ^2^ Dyad*Rater	30.2%***	30.2%***	30.2%***	30.2%***	5.3%*	3.8%	0.0%	3.7%*
σ^2^ Residual Error	32.5%	27.0%	68.8%	18.2%	41.0%	44.6%	68.4%	32.0%

*Note*: Each column represents the respective CAID parameter, whereas each row represents the source of influence on CAID parameter variance. Possible sources of variance included Dyads (n = 61), situations (n = 4), rater (n = 10), and their relevant interactions. Variation proportions within each column sum to 100%, within rounding error. Significance for random effects were determined with Likelihood-Ratio tests within each parameter using lmerTest package in R

(* p ≤ .05

** p ≤ .01

*** p ≤ .001). Fox et al. [16] G study results are reported for ease of comparison.

#### Mean-levels of warmth and dominance

The general pattern of results suggests that the CAID parameters were influenced by multiple systematic sources. Residual error accounted for 6–69% of the variance across CAID parameters. The main influences on mean warmth were the person (14.9%), dyad (19.9%), rater (10%), and the dyad x rater interaction (14.3%). Other sources of influence had minimal impact. Main influences on mean dominance included adolescent role (45.5%), the person (20.6%), and the person x rater interaction (9.8%). Again, other sources of influence had minimal impact.

These results support that warmth and dominance may be more trait-like for some individuals, at least within the context of the observed interactions and dyad. They also suggest that certain raters may be biased towards observing warmth or dominance in certain dyads. Finally, they suggest that characteristics of individuals within the dyad, like whether the individual is in the parent or adolescent role, may influence individual levels of warmth or dominance. Moreover, some dyads tend to exhibit more or less warmth than others. Consistent with Fox et al. [16], the dyad was the largest influence on mean-levels of warmth, followed by the dyad x rater interaction and individual differences of people. Also similar across the two studies, dominance was heavily influenced by individual differences. However, dominance was most heavily influenced by the role of adolescent versus parent in our sample. This is perhaps unsurprising given the built-in power dynamic in parent-adolescent relationships that is not present in romantic partnerships [1, 11].

#### Variability in warmth and dominance

The factors that influenced variability in warmth and dominance were similar to those that influenced mean scores. The rater (23.5%) and the dyad x rater interaction (22.6%) influenced variability in warmth whereas the rater (41.3%), the dyad x rater interaction (14.2%), and the person x rater interaction (9.8%) predicted variability in dominance. These data suggest that deviations from typical warmth and dominance were strongly influenced by how the rater perceived certain individuals and dyads. This is consistent with Fox and colleague’s [16] findings that the rater, followed by a number of interactions with the rater (i.e., situation, dyad), had the largest influence on both warmth and dominance variability.

#### Shape

In terms of shape, or the intra-individual correlation between warmth and dominance, only the person x rater interaction (15.4%) and the dyad x rater interaction (11.1%) significantly influenced shape. This suggests that some raters were more or less likely to observe shape based on certain individuals and dyads. Influences on shape in our study were less consistent with Fox et al. [16], who found a strong effect of the person x situation interaction whereas shape in our study was influenced by the person x rater and dyad x rater interactions. It seems that shape in parents and adolescents is less influenced by individual differences and the situation and varies as a function how raters perceive certain individuals and certain parent-adolescent relationships.

#### Complementarity

Complementarity was assessed at the dyadic level, therefore, only the dyad, situation, and rater variable could influence variation in complementarity estimates. The dyad (30.2%) and dyad x rater interaction (25.7%) had strong and significant influences on mean-level warmth complementarity, suggesting that some parent-adolescent dyads tended to be more congruous on their warmth compared to others, and some raters viewed parent-adolescent dyads as exhibiting more warmth complementarity on average compared to other raters. The dyad x situation interaction (13.6%) had a strong and significant influence on moment-to-moment warmth complementarity, indicating that some parent-adolescent dyads showed more warmth congruity during some situations.

The dyad x rater interaction (35.8%), rater (14.9%), and dyad (9.9%) had significant influences on mean-level dominance complementarity. These results suggest that some raters tended to rate parent-adolescent dyads as more dominance congruent on average compared to other raters, some raters perceived more dominance congruity across the board on average, and some dyads exhibited more dominance congruity than others on average. The dyad (27.5%), rater (22.6), and dyad x situation interaction (15.5%) had a strong and significant influence on moment-to-moment dominance complementarity. These results suggest some parent-adolescent dyads exhibited more moment-to-moment reciprocity compared to other dyads, and some raters viewed more momentary dominance complementarity compared to other raters. Additionally, dyadic dominance congruence was stronger in some situations compared to others.

Our results suggest that some dyads naturally exhibited more warmth complementarity on average than others, that dominance complementarity is more readily observed by some raters than others, and that some raters were more likely to rate certain dyads higher on warmth and dominance complementarity than others. The main effect of rater on dominance but not warmth complementarity could be explained by the relatively smaller amount of overall variance in warmth than dominance for both teens and parents. This strong dyad effect, as well as a rater effect on mean-level dominance complementarity, is consistent with Fox et al.’s [16] findings. However, our findings diverge from Fox and colleagues in that they also found a strong situation and couple x situation interaction effect on warmth whereas we found a strong rater and rater x dyad interaction effect on mean-level warmth and dominance. Perhaps parent and adolescent responses to one another are more subject to an observer’s bias compared to married couples. For instance, an observer may hold stronger preconceived notions about or more readily recognize certain parenting behaviors, like discipline, compared to more ambiguous behaviors between romantic partners, like using conflict resolution skills.

In terms of moment-to-moment warmth complementarity, consistent with Fox et al. [16], only the dyad x situation interaction had a substantial influence on estimates. This suggests that some parent and teen dyads varied in their warmth reciprocity depending on the topic discussed as the situation unfolded. This was also the case for moment-to-moment dominance complementarity. Other influences on moment-to-moment dominance complementarity included the dyad and the rater. These results are largely consistent with Fox et al. [16] as they also found a large dyad and dyad x situation effect on momentary dominance complementarity. Some raters were more likely to perceive submissiveness in response to dominance, or vice versa, and some dyads were more likely to demonstrate dominance complementarity (i.e., higher dominance leads to higher submissiveness) than other dyads as situations unfolded.

### D study results

The D study estimates are used to assess between and within-person reliability across situations using variances from the G study (*see* Table 5). Following recommended guidelines for interpreting intraclass correlations of observational data [57, 58], we interpreted reliabilities less than .40 as poor, .41 to .59 as fair, .60 to .74 as good, and .75 to 1.0 as excellent.

**Table 5:** Between- and within- person and dyad consistency of interpersonal behaviors (D study analyses).

***Current Study***		**Indices of Individual Behavior**					**Indices of Dyadic Complementarity**			
		Average		Variability		Shape	Mean-Level		Moment-to-Moment	
	*Estimate of Consistency*	*Warm*	*Dom*	*Warm*	*Dom*	*Warm-Dom*	*Warm*	*Dom*	*Warm*	*Dom*
R_1F_	Between-person reliability of observations *within a situation*	.85	.94	.43	.53	.38	.82	.74	.25	.87
R_KF_	Between-person reliability of observations *across all situations*	.98	.99	.86	.88	.78	.98	.95	.72	.98
R_1R_	Between-person reliability of observations of *different situations*	.58	.93	.23	.34	.23	.81	.61	.24	.71
R_C_	Within-person reliability of *change across situations*	.75	.93	.50	.23	.65	.66	.72	.66	.89
***Fox et al*. *(2021)***		**Indices of Individual Behavior**					**Indices of Dyadic Complementarity**			
		Average		Variability		Shape	Mean-Level		Moment-to-Moment	
	*Estimate of Consistency*	*Warm*	*Dom*	*Warm*	*Dom*	*Warm-Dom*	*Warm*	*Dom*	*Warm*	*Dom*
R_1F_	Between-person reliability of observations *within a situation*	.69	.90	.41	.25	.30	.70	.48	.26	.76
R_KF_	Between-person reliability of observations *across all situations*	.96	.99	.87	.77	.79	.96	.90	.78	.97
R_1R_	Between-person reliability of observations of *different situations*	.37	.84	.22	.08	.18	.64	.37	.22	.60
R_C_	Within-person reliability of *change across situations*	.75	.92	.36	.57	.84	.84	.80	.70	.86

*Note*: Consistency in indices of complementarity reflect estimates of between- and within-*Dyad* influences, rather than between- and within-*person* influences.

#### Mean-levels of warmth and dominance

Reliability estimates of the between-person reliability of observations within a situation (R_1F_) suggest that raters provided excellent ratings of mean warmth (.85) and dominance (.94) within a given situation. Mean ratings of warmth (.98) and dominance (.99) aggregated across all raters and situations (R_KF_) were also excellent. When considering how scores form a single situation generalized across different situations (R_1R_), mean warmth was fair (.58) but mean dominance was excellent (.93). This suggests that single assessments of dominance provided reliable estimates of a person’s general tendency to be dominant, but this was less true for warmth. Lastly, within-person reliability estimates of change across all situations (R_C_) were excellent for both warmth (.75) and dominance (.93). The majority of estimates fell in the excellent range, and this overall pattern of results suggests that a person’s general tendency to be warm or dominant, relative to others, can be reliably estimated from a single interaction using the CAID system with the possible exception of average levels of warmth across differing situations.

#### Variability in warmth and dominance

Averaged scores across all raters in a single situation (R_1F_) provided fairly reliable estimates for warmth (.43) and dominance (.53). Reliability was excellent for variability estimates of warmth (.86) and dominance (.88) averaged across all situations (R_KF_). Reliability for variability estimates across different situations (R_1R_) was poor for warmth (.23) and dominance (.34). For within-person change across situations (R_C_), warmth variability estimates were fairly reliable (.50) whereas dominance variability estimates had poor reliability (.23). This pattern of results suggest that variability estimates were more reliable when averaged across all situations at the between and within-person level compared to between-person estimates within and across different situations. When comparing variability reliability estimates to mean-levels of warmth and dominance, variation in these interpersonal dynamics appears more difficult to capture consistently within a given situation or individual, but reliability is comparable when averaging across all situations (R_KF_). The rater had the largest influences on variability estimates, whereas the person and dyad had the largest influences on mean ratings. In other words, measuring variation seemed to be driven more by the rater whereas mean levels depended more on the observed participants, suggesting that raters may be perceiving changes in variability rather than detecting real changes.

#### Shape

Estimates for interpersonal shape had poor reliability within a situation (R_1F_ = .38) and across different situations (R_1R_ = .23). Shape estimates were more reliable across all situations at the between-person level (R_KF_ = .78) and for change at the within-person level (R_C_ = .65). Similar to variability estimates, shape parameters were more reliable across all situations (i.e., more data points included) at the between and within-person level. Notably, shape was influenced by several rater interactions (rater x person and rater x dyad), suggesting some raters exhibited greater difficulty in detecting joint changes in warmth and dominance for some individuals and dyads compared to others.

#### Complementarity

Mean-level complementarity reliability estimates within a single situation (R_1F_) were good for dominance (.74) and excellent for warmth (.82). Moment-to-moment dominance complementarity also had excellent reliability (.87), but warmth estimates had poor reliability (.25). Similar to estimates of individual behavior, complementarity estimates averaged across all raters and situations (R_KF_) had excellent reliability for warmth (.98) and dominance (.95). This was also true for moment-to-moment estimates of dominance (.98), but estimates for moment-to-moment warmth complementarity were somewhat less reliable (.72) than the average. Reliabilities for estimates of between-dyad complementarity in different situations (R_1R_) were excellent and good for warmth (.81) and dominance (.61), respectively. When assessing moment-to-moment estimates of complementarity in different situations between dyads, however, warmth had poor (.24) and dominance had good reliability (.71). Lastly, reliability for within-dyad mean estimates of change across situations (R_C_) was good for both warmth (.66) and dominance (.72). Moment-to-moment reliability was also good for warmth (.66) and was excellent for dominance (.89).

Given poor warmth reliability on individual-level estimates in single situations (R_1F_ and R_1R_), it is unsurprising that we observed poor reliability for moment-to-moment warmth complementarity in single situations. Rater and dyad effects (and their interaction) were among the strongest influences on individual-level warmth estimates whereas the dyad and the dyad x rater and dyad x situation interactions were among the among the strongest predictors of dyad-level warmth complementarity. This suggests that some raters were less reliable at detecting true levels and changes in warmth and that it was difficult to detect levels and changes in warmth in some dyads compared to others.

Overall, our general pattern of results from our D study largely reflects those of Fox et al. [16]. Both studies found that CAID parameters tend to be most reliable when assessed across multiple situations by multiple raters. Estimates of single assessments, however, still provided some reliable estimates and are still valuable. Given that, similar to Fox and colleagues [16], variability estimates tended to be the least reliable, future studies should examine whether increased situations, raters, and sample sizes improve reliabilities of variability estimates. Similarly, reliability tended to be lower for warmth estimates. Given that warmth variability was limited in general during interactions, changes in stable patterns of warmth behaviors may be more difficult to detect. Fox and colleagues [16] similarly found lower reliability for warmth estimates, suggesting this is not unique to parent-adolescent dyads. Since highly consistent patterns in reliabilities were found between Fox et al. [16] and the current study, with only a few exceptions, the CAID system appears to be a robust observational assessment tool that can be used across different types of relationships.

## Limitations and future directions

The pattern of results from the GT analyses indicated that the CAID can assess individual and dyadic parameters reliably for a single situation but reliability increases with additional situations and likely improves with additional raters. Although multiple situations and multiple raters may increase CAID reliability, these steps would also undermine the feasibility of using the CAID in research and clinical practice. Additional work that seeks to expedite the scoring process of the CAID, such as the use of automated scoring procedures or increased training in detecting warmth, is needed to enhance the feasibility of the CAID in research and clinical practice. Relatedly, given that robust rater effects were observed, future work should seek to test whether certain rater characteristics influence reliability or the strength of CAID parameters to predict outcomes. For example, a rater’s gender or race may affect coding of participants with different identities or influence perceptions of what behaviors constitute warmth or dominance. Moreover, parent and adolescent’s gender and race may also need to be considered. We are unaware of any work examining potential rater race and gender influences on CAID parameters, and this would be important to consider in future research. Additionally, we utilized a community sample, where parent-adolescent relationships are generally positive [59]. Future work should seek to validate this methodology in clinical samples, where parent or adolescent psychopathology may influence how these interpersonal dynamics unfold [60]. Last, although the number of data points used was large, the sample size for the current study was relatively small and all but one participating caregiver was female. Future work should seek to assess the reliability of the CAID for parent-adolescent interactions using larger samples and both male and female caregivers.

## Conclusion and clinical implications

Our findings support using the CAID system and GT analyses to improve researchers’ understanding of how parent and adolescent dynamics unfold over time and across various discussion topics or contexts. We identified several important factors that influence interpersonal behavior ratings and the circumstances in which these rating can most reliably be applied. While there were some notable differences unique to parent and adolescent dyads, our findings are mostly consistent with other work done with romantic couples [16], suggesting the CAID system methods can reliably generalize across different kinds of relationships.

CAID could be used to assess parent-adolescent dynamics with an eye toward improving dyadic functioning in a clinical context. In doing so, a few interesting considerations from our findings are worth noting. First, the strong rater effect has interesting clinical applications as some therapists may perceive more or less variations in families as these interpersonal dynamics unfold in session. Many family therapies view the parent-child relationship as the core mechanism of change, and CAID could be a useful tool in quantifying a clinician’s bias when observing their interactions (i.e., the strength of the rater effect) and its impact on outcomes of interest (e.g., post-treatment symptom severity or rapport building). A number of empirically-supported parent-child interventions utilize standardized behavioral observation assessments to monitor intervention effectiveness and predict meaningful clinical outcomes [61]. One advantage CAID has over existing parent-child interaction coding schemes is its utility in various kinds of dyads (e.g., adolescent versus child and caregiver). As work continues to improve the CAID coding system, it may also be possible to improve clinical assessment procedures through training student clinicians in the use of CAID. Second, some parent-adolescent dyads exhibited varying levels of warmth and dominance on average but also varied more drastically from those averages compared to other dyads. These findings have important intervention implications as families could be identified as at greater risk for poor outcomes based on average levels of these interpersonal dynamics (e.g., low warmth and high dominance) as well as how likely parent-adolescent dyads are to deviate from these averages in the moment when discussing certain topics (e.g., some topics may elicit greater dominance from a parent compared to others) [12]. Last, although shape has received little attention in the literature [36], it has potentially important implications for parenting interventions. The parenting styles literature supports a robust protective effect for an authoritative parenting style (i.e., high warmth and dominance) on adolescent substance use [62], but the literature lacks fine grained assessment measures of how parenting styles operate and change from moment-to-moment. The CAID system allows us to better understand how shape operates across different situations and unfolds during interactions with adolescents over time. This could lead to improved parenting interventions that explicitly target dynamics like shape that help parents foster an authoritative style when interacting with adolescents.

## Supporting information

S1 Appendix Alcohol and cannabis discussions. (DOCX)

S2 Appendix Joystick manual. (DOCX)

S1 Table Variance parameters. (DOCX)
