# Correction: Variance Decomposition of the Continuous Assessment of Interpersonal Dynamics (CAID) system: Assessing sources of influence and reliability of observations of parent-teen interactions

**DOI:** 10.1371/journal.pone.0333912

**Published:** 2025-10-06

**Authors:** The PLOS ONE Staff

Table 4 contains repeated values of ‘30.2%***’. Please see the correct Table 4 here.

The publisher apologizes for the error.

**Table 4:** Partitioning of Total Variance in Dyadic-Level Kinspersons’ Interpersonal Behaviors (G Study Analyses).

	Indices of Dyadic Complementarity – Current Study				Indices of Dyadic Complementarity – Fox et al. (2021)			
	Mean-Level		Moment-to-Moment		Mean-Level		Moment-to-Moment	
*Source of Variance*	*Warm*	*Dom*	*Warm*	*Dom*	*Warm*	*Dom*	*Warm*	*Dom*
σ^2^ Dyad	30.2%^***^	9.9%***	3.6%	27.5%***	22.3%***	9.4%***	6.0%***	24.3%***
σ^2^ Situation	0.3%	3.4%***	0.2%	6.6%***	1.0%	1.3%	0.0%	6.0%***
σ^2^ Rater	4.6%^***^	14.9%***	5.6%^***^	22.6%***	7.1%***	18.3%***	5.2%**	11.8%***
σ^2^ Dyad*Situation	6.4%^***^	7.1%***	13.6%^***^	15.5%^***^	21.5%***	17.6%***	15.7%***	19.3%***
σ^2^ Rater*Situation	0.4%	1.9%***	0.0%	0.8%**	1.8%***	4.9%***	4.6%***	3.0%***
σ^2^ Dyad*Rater	25.7%^***^	35.8%***	8.2%^***^	8.8%^***^	5.3%*	3.8%	0.0%	3.7%*
σ^2^ Residual Error	32.5%	27.0%	68.8%	18.2%	41.0%	44.6%	68.4%	32.0%

*Note:* Each column represents the respective CAID parameter, whereas each row represents the source of influence on CAID parameter variance. Possible sources of variance included Dyads (n = 61), situations (n = 4), rater (n = 10), and their relevant interactions. Variation proportions within each column sum to 100%, within rounding error. Significance for random effects were determined with Likelihood-Ratio tests within each parameter using lmerTest package in R (* p ≤ .05, ** p ≤ .01, *** p ≤ .001). Fox et al. [16] G study results are reported for ease of comparison.
