# Supplementary material for: Variance Decomposition of the Continuous Assessment of Interpersonal Dynamics (CAID) system: Assessing sources of influence and reliability of observations of parent-teen interactions
Source: PLoS One. 2023 Oct 18;18(10):e0292304. doi: 10.1371/journal.pone.0292304 (PMC10584132; doi:10.1371/journal.pone.0292304)
Supplement: S1 Appendix — (DOCX) [file pone.0292304.s001.docx]

**Supplemental Appendix 1: Alcohol and Cannabis Discussion Prompts**

**Task Instructions**: “Families have different views and beliefs about alcohol use. For the next 9 minutes, I would like the both of you to talk with each other about your family’s beliefs and attitudes about alcohol use. We have a list of some issues for you to discuss, and I would like you to come to an agreement about them. After you have said all you have to say about an item, you can move on to another item. …It is not important to get through all the items. We do not want you to feel rushed. If you want to take the whole time talking about one or two of the items that would be fine.”

**Alcohol Topics List**

It is acceptable/unacceptable in your family for:

- Adults to get a little tipsy now and again in front of their children
- Kids to sip and taste alcohol with adult supervision
- Parents to tell stories about their own drinking to their children
- Kids to make their own decisions about alcohol and leave parents out of it
- Adults to drink alcohol whenever they want
- Kids to drink unsupervised with their friends on Friday/Saturday nights.
- Parents to punish their children if they are caught drinking alcohol without permission
- Kids to fetch, pour, or serve drinks to others in the home
- Parents to get angry if their child is caught drinking without permission
- Kids to have an alcoholic drink at a family event
- Parents to discuss with their children what to do if offered alcohol at a party
- Kids to have an alcoholic drink with their friends in the family home under adult supervision
- Kids to be at a party where their friends are drinking

**Task Instructions**: “Families have different views and beliefs about marijuana use. For the next 9 minutes, I would like the both of you to talk with each other about your family’s beliefs and attitudes about marijuana use. We have a list of some issues for you to discuss, and I would like you to come to an agreement about them. After you have said all you have to say about an item, you can move on to another item. …It is not important to get through all the items. We do not want you to feel rushed. If you want to take the whole time talking about one or two of the items that would be fine.”

**Marijuana Topics List**

It is acceptable/unacceptable in your family for:

- Adults to get a little high now and again in front of their children
- Kids to try marijuana with adult supervision
- To keep marijuana in the house
- Parents to tell stories about their own marijuana use to their children
- Kids to make their own decisions about marijuana use and leave parents out of it
- Adults to use marijuana whenever they want
- Kids to use marijuana unsupervised with their friends on Friday/Saturday nights.
- Parents to punish their children if they are caught using marijuana without permission
- Kids to fetch or prepare marijuana for others in the home
- Parents to get angry if their child is caught using marijuana without permission
- Kids to use marijuana at a family event
- Parents to discuss with their children what to do if offered marijuana at a party
- Kids to use marijuana with their friends in the family home under adult supervision
- Kids to be at a party where their friends are using marijuana
