# Supplementary material for: Variance Decomposition of the Continuous Assessment of Interpersonal Dynamics (CAID) system: Assessing sources of influence and reliability of observations of parent-teen interactions
Source: PLoS One. 2023 Oct 18;18(10):e0292304. doi: 10.1371/journal.pone.0292304 (PMC10584132; doi:10.1371/journal.pone.0292304)
Supplement: S2 Appendix — (DOCX) [file pone.0292304.s002.docx]

**Joystick Method:**

**Data and Procedures Manual**

2019

**Table of Contents**

[Video Breakdown 3](#_Toc19830726)

[First Meeting 4](#_Toc19830727)

[Overview of Training Session 5](#_Toc19830728)

[Data Collection & Management 9](#_Toc19830729)

[Research Using The Joystick Method 14](#_Toc19830730)

[Appendix A: Script for Learning to Use the Joystick 16](#_Toc19830731)

[Appendix B: Script for Introducing Joystick Coding 18](#_Toc19830732)

[Appendix C: Joystick Issues That Trainees May Have 20](#_Toc19830733)

[Appendix D: Lab Cheat Sheet 22](#_Toc19830734)

*All CAID coding procedures and training materials were adapted from training materials and scripts originally developed by Pamela Sadler and Erik Woody (Sadler et al., 2009; Sadler & Woody, 2023).

# **Video Breakdown**

6 weeks, 2 computers

*Without Warm Up Videos*

- 1 hour per dyad
- 5 raters per video
- 60 videos = 300 person hours
- 10 raters total = 30 hours per person
- Each person has to average 5 hours per week in 1 hour chunks

*With Warm Up Videos*

- 1.5 hour per dyad
- 5 raters per video
- 60 videos = 450 person hours
- 10 raters total = 45 hours per person
- Each person has to average 7.5 hours per week in 1.5 hour chunks

# **First Meeting**

The first meeting will happen with the RAs at the beginning of the quarter. Below are things to review with them during that meeting.

**Agenda**

- Expectations
- Lab Related Things
- Review videos

**Specifics**

- Expectations
  - Don’t get back to us in a week, then they are no longer in the lab
  - By x date each time you have to have schedule complete
    - They will get 1 reminder
  - If you haven’t responded in 2 days after reminder, you’re no longer in the lab
- Lab Related Things - Review
  - 7.5 hours of video coding per week in 1.5 hour chunks
    - Can do longer than 1.5 hours but must take breaks
  - RA Log Spreadsheet
    - Will be coding dyads of parents and teens talking in 4 videos – letters tell them which participant to code first
    - Replace who they code with an X
    - Second section of words is order to code videos in
    - Comments column
      - Any aspects of the video that can interfere with coding (e.g., if the video is hard to hear, sound cuts out momentarily from X to Y time, research team interjects, the video zooms, etc.).
  - [Calendar](https://calendar.google.com/calendar?cid=Z2I1OHUyYzF0YWd1bW84NGVkcDFwMm5jNTBAZ3JvdXAuY2FsZW5kYXIuZ29vZ2xlLmNvbQ) to sign up for coding
    - First and last name
    - Can color code themselves if they like
    - If you can’t make your time anymore, remove yourself from the calendar!
      - That way if people end up having some free time, they can stop by and code
      - People on the calendar have priority though
    - Sign up early! So you’re not squeezed at the end!
  - 2 computers only
  - Jordan will send out video assignments at the beginning of the 2 week period and they can also check the spreadsheet
  - Weekly meetings Fridays 9 – 10:30 am in 1314 SSH
- Look at videos from training
  - Highlight places where people’s codes really differed
    - Discuss how people interpreted actions in those places

# **Overview of Training Session**

- Running the Training
  - “Interrupt me with questions at any time!”
  - Go slowly and explicitly for every point here
  - Check for confusion after each point
  - Have them repeat points back
  - Reference the cheat sheets as much as possible so they associate the steps they’re learning with looking at it
- In the Room
  - Equipment
    - Computer they will use
      - 2 laptops available
      - Please don’t move them
    - Headphones
    - Joystick
      - Adjust it so it is comfortable to you and use your dominant hand
      - You can rest your elbow on the desk or chair
  - Open the computer, press the power button, and log on using the cheat sheet on the wall
  - DARMA will open automatically (it may take a second)
  - Open Chrome on the laptop and go to the URL on the cheat sheet
    - RA Log Spreadsheet
      - Will be coding dyads of parents and teens talking in 4 videos – letters tell them which participant to code first
      - Replace who they code with an X
      - Second section of words is order to code videos in
      - Comments column
        - Any aspects of the video that can interfere with coding (e.g., if the video is hard to hear, sound cuts out momentarily from X to Y time, research team interjects, the video zooms, etc.).
        - If there is no sound in a whole video, they should make a note in the comments section for that dyad and then skip coding the video
      - 7.5 hours of video coding per week in 1.5 hour chunks
        - Can do longer than 1.5 hours but must take breaks
      - Every time you will check the spreadsheet for what videos you are supposed to code, the order to code the participants in, as well as the order to code the scenes in
      - They should recode the warm up videos if they are assigned to the ones from training
- Have them watch the Powerpoint in the “Training Powerpoint” folder
  - Tell them it’s not psychotherapy videos
  - And that the axis is going to be “separate”
- *Appendix A: Script for Learning to Use the Joystick*
  - Where to find the videos
    - “Coding Project 2019” folder that is pinned on the left side of the file window
    - The video folders in there are split up by scene, each video labeled with the dyad number
    - They cannot keep or distribute videos
  - When to take breaks
    - You should be watching all the videos for 1 participant and then switching
    - Try to get through a whole dyad and then take a break
      - If you can’t do all of them without taking a break, try to get through a whole participant in a dyad (all 4 videos)
    - Do not stop a video in the middle, always code the whole video in 1 sitting
      - DARMA does not record the coding until the end of the video
  - Use the Dyad 3 warm up video for demonstrating the joystick and having them do preliminary practice
- **It is extremely important to ensure that you are coding the correct dyad as well as the correct scene**
- Have the coder try coding the 4 training videos (dyads 4, 7, 27, 33) one at a time
  - *Appendix B: Script for Introducing Joystick Coding*
  - Give feedback in between
    - *Appendix C: Joystick Issues That Trainees May Have*
    - After every video they code, pull up the Review Ratings pane and compare their codes to the reliability code for that video
    - Click “Analyze Ratings” and look at their means, SDs, and Average-Score Agreement ICCs for each axis
      - “This is what we will be discussing in our weekly meetings too. We will be looking at everyone’s codes compared to each other in terms of the means, SDs, and Average-Score Agreement ICCs for both axes.”
    - Highlight spots where they differentiated from the reliability coder and discuss why
      - Discuss what’s happening in the scene at that point
        - “We will break down scenes in this way during meetings too and will discuss the places where people had the most different reactions to what’s happening in the scene.”
      - Which axis in their code is different? Why do they think they coded that spot differently?
  - To advance from one video to the next, they need on average, an Average-Score Agreement ICC >= 0.4 on both axes for both participants when compared to the reliability coder (Chris’ and Jordan’s codes for the training videos averaged). Additionally, they cannot have any Average-Score Agreement ICC < 0.3 to pass
    - For example, for the parent x axis they could have a 0.35 ICC if the parent y axis is a 0.45 ICC because that averages out to 0.4. However, they could not pass with an ICC of 0.51 on the x axis if the y axis is an ICC of 0.29 because 0.29 is too low
    - If they don’t hit these requirements, have them recode the video and then discuss again
    - Have them recode the video until they get that agreement or you run out of time
      - You should be having a pretty in depth conversation about the circumplex and the participant’s actions by the time an RA is recoding a video for the third or fourth time
      - If they’re having trouble, is there a certain place where they don’t understand that they should be coding something a specific way? Do they need to take a short break? Try to understand what the sticking points are for their coding
    - If the RA does not get to all of the training videos, they should reschedule with the trainer in the next couple of days to meet and code again
  - If DARMA freezes at all during the training, have them reference the cheat sheet on the wall and quit DARMA / start it up again
- Naming & saving the files
  - Each output coding file will have the form (no spaces): Dyad#_ Scene_Target (P or T)_Coder initials.csv.
    - For example, if Jordan codes the teen from dyad 4 of the parent teen warm up interaction, she would name the file: 04_WarmUp_T_JV.csv.
  - Show where to save the files
    - “Coding Project 2019” folder and then their named folder inside
  - Reference the cheat sheet
    - “The cheat sheet on the wall is to help you remember what to do every time”
- Show how to delete coding files, if needed
  - Do this CAREFULLY!
- Helpful Reminders
  - Get all your stuff set up before you start coding
  - “Move the joystick!”
  - “Much better to take breaks than to lose attention”
  - “I will see in the graphs if you’re not paying attention”
  - Go with whatever your instinct is, don’t over think it
  - Focus on just 1 person at a time
  - Try to code both people in 1 sitting
- “After each joystick assessment, remember to complete the coding log, filling in which participant was coded, and if there were any notable issues”
- **Have them talk you through the whole process using the cheat sheets on the wall**
  - To check for understanding and also to prep them for doing the process on their own with only the cheat sheets
- Out of the Room
  - Weekly meetings Fridays at 9 – 10:30 am in 1314 SSH
  - Calendar to sign up for coding
    - Share with them during training
    - First and last name
    - Can color code themselves if they like
    - If you can’t make your time anymore, remove yourself from the calendar!
      - That way if people end up having some free time, they can stop by and code
    - Sign up early! So you’re not squeezed at the end!
  - Jordan will send out video assignments at the beginning of the 2 week period and they can also check the spreadsheet

**Training Videos**

- Dyads 3, 4, 7, 27, 33 Warm Up Videos
  - 4 minutes each

# **Data Collection & Management**

This section describes how coders are assigned video interactions, how they record and save the data, how to monitor data collection, and how to prepare for regular coder meetings.

**Coding Assignments**

We ask RAs to commit 10 hours per week to our lab, 8.5 hours scheduled with built in leeway time. We expect them to code 7.5 hours per week and to also attend weekly 1 hour coder meetings. Coding hours should include time to set up each video, make comments regarding videos, save data files, and some time for breaks. For example, if we are coding 31 minute videos, RAs would be expected to code 1 video every 1.5 hours (31 minutes to code each individual = 1 hour 2 minutes, plus ~10-15 minutes to set up, save data, and make notes to other coders when applicable, plus ~5-10 minutes to take a break). Thus, a 7.5 hour coding assignment would consist of 5 videos.

Depending on the project, rather than assigning 7.5 hours to code for each week, it often makes more sense to ‘work backwards’ and first determine how many hours of coding the project will require and to then allot RAs the appropriate amount of time to complete the assignment. For instance, if we have 60 dyads that each interact for 31 minutes and we allot 1.5 hours per video, it will take 450 hours to complete the coding assignment. At a rate of 7.5 hours per week, 10 coders can code these videos in 6 weeks. However, we have found that coders can begin to lose track (or get really behind) on long assignments. For this reason, it’s best to break up any assignments that will take longer than one month into smaller assignments. So for this example, you could break up the 45 hours per coder over 6 weeks into smaller parts (e.g. giving out 10 videos to code every 2 weeks at a rate of 7.5 hours per week).

We give RAs autonomy regarding how they complete assignments. They can code any time the building is open (between 7 am and 7 pm on weekdays). Further, they can choose how to allocate their coding time. For instance, some RAs prefer to code more frequently for shorter blocks of time (e.g., 3x/wk for 2.5hrs) whereas other RAs prefer to code less frequently for longer blocks of time (e.g., 2x/wk for 3.75hrs). Although RAs are encouraged to maintain a pace of 7.5 hrs/wk, they also have the freedom to code more some weeks and less others. For instance, if we give a 2 week coding assignment and an RA has a particularly busy week during the second week, they could choose to code 11.25 hours the first week and 3.75 hours the second week. As long as RAs complete their assignments on time and are attentive and reliable when coding, they can create their schedules as they wish.

We give all RAs access to our shared *Hopwood Lab Coding Calendar* Google calendar. Although project managers should add coder meetings to the schedule, the main purpose of the calendar is to allow RAs to reserve coding times. This prevents multiple people from coming in to code at the same time, only to have to leave because the computers are already occupied. Because we currently have two computers, two RAs can sign up to code at any given time.  To sign up for a time, RAs simply write their name in the block of time they want to code. RAs are permitted to code even when they are not signed up (e.g., if a class ends early and they have some spare time). However, if two coders are already signed up during that time then those coders have priority.

A minimum of 5 RAs should code each dyad. RAs should always code both participants in a dyad to ensure that observed differences between dyad members are not an artifact of differences between coders. The videos should be randomized across coders and for the Buffalo project we ensured that all coders were given an equal number of videos to code. Also we made sure that all RAs had a similar number of videos in common with all other RAs.

We use Google Drive spreadsheets to create coding assignments and share them with RAs. We use Drive instead of Excel because of the editing abilities for multiple people at once in Drive. This is important since we often have 2 RAs coding at the same time, both of whom need to edit the document when they’re done coding. Specifically, after coding a video, the RA replaces the letters indicating which participant to code with an ‘X’ to indicate that they have coded this video. RAs will also sometimes make notes on any aspects of the video that can interfere with coding (e.g., if the video is hard to hear, cuts out momentarily from X to Y time, etc.).

We always create two Google Drive spreadsheets: a *master file* and a *shared file*. The master file is created first, and then copied to create a shared file. The shared file will be edited by RAs as they complete the assignment. The master file will not be edited and should only be owned by the project manager to ensure that RAs don’t accidentally make changes to it. This file indicates the order in which individuals in a dyad were coded across RAs.

**Coding Assignment Files**

*Master File – RA Coding Log*

For this data collection, we have a total of 10 coders, 60 dyads, and 240 videos. Each RA will code half of the videos and each video will be coded by 5 RAs.

- Column A of this file lists the *dyad number* for all dyads in the study
- Columns B-K list the names of each coder in the study and the videos they should code as well as the order to code the participants and scenes in
- Column L is for relevant *comments*
- Column M lists the sum of coders assigned to each dyad. The ‘countA’ formula function is used to sum the number of coders assigned in each row. For instance, for the first dyad, this formula reads: =COUNTA(B2:K2). After creating this formula for the first row, you can click on the bottom right of the cell and simply drag down to insert the same function in all the rows

After creating these columns, coders should be assigned to videos (you can use a sampling function in R). The amount of videos each RA had in common with every other RA should be checked (R code was written for this for the Buffalo project). The optimal number of videos in common for the Buffalo project is 13-14. The assignments then most likely will need to be tweaked by hand and adjusted so that every RA has 12-15 videos in common with every other RA. Also, because you will be meeting weekly, it makes sense to assign the videos in sets such that each RA codes ~10 videos for dyads 1-20, 21-40, and 41-60. So that there are multiple RA codes to look at for each video for each meeting.

Once RAs are assigned to videos, run the assignments through R code that randomly generates the order in which RAs should code the people in the dyad as well as the participant scenes. Check the dyad assignments to make sure they are balanced such that there are 3 RAs coding one order of participants and 2 the other. For this data collection, P = coding the parent and T = coding the teen.

At the bottom of the coding log (row 62), is the same ‘countA’ function used for column M to count the number of dyads assigned to each coder. It is important to ensure that all RAs are assigned an approximately equal number of dyads to code. ‘countA’ was also done for each video chunk (1-20, 21-40, and 41-60) to make sure video assignments were spread out evenly.

*Shared File*

After creating the master coding assignment file, you can copy this file in Google Drive by clicking ‘file’ -> ‘make a copy.’ The default naming procedure in Drive is for this file to save as ‘copy of [original file name].’ Instead of using this format, you can rename the master file as ‘[name of coding assignment] – Master Copy.’ So in this example, these two files were named ‘RA Coding Log’ and ‘RA Coding Log – Master Copy.’ You can delete the ‘sum’ column and row in the shared file. Then click the ‘share’ button in Google Drive to share the file with all relevant RAs. Another sharing option is to make a bit.ly link for the RA Coding Log so the RAs can find / remember it more easily.

RAs make two primary edits to the shared file:

- After coding a dyad, the RA replaces the letters indicating that dyad should be coded (PT or TP in this example) with an ‘X’ to indicate they have coded this dyad. We encourage RAs to code both members of a dyad during a coding session, but if this is not possible for some reason, they can indicate that they have coded one but not both individuals by replacing the letter for the individual they coded with an ‘X’ but keeping the letter for the individual they did not code. For instance, if an RA coded the parent but not the teen for a dyad, they could replace ‘PT’ with ‘XT.’
- RAs can make notes to one another about the videos in the ‘comments’ column. Notes often include interruptions during the task, difficulty understanding one or both individuals, or problems with video quality. In most cases, the videos should still be coded, but it can be useful for other RAs to know what to expect prior to coding a video. If RAs are unsure about whether a video should be coded for any reason, they should email and ask the project manager (e.g., in one video, one couple begins speaking another language for ~30 seconds towards the end of the video. We chose to keep coding based on nonverbals during this time).

**Cheat Sheet**

Printing out a cheat sheet with the coding steps on it is helpful for RAs to remember how to open up the files and get coding. The ones we had for the Buffalo study are in Appendix D.

**Software and Saving Data Files**

DARMA is the application we use to joystick code. When the laptops are opened up, DARMA opens automatically. To find it yourself for any reason, you can search for it in the search bar or click on the Windows icon on the bottom left to find it.

[Here](https://github.com/jmgirard/DARMA/wiki) is the documentation for DARMA that reviews how to use it. When you open the application, there are two options: Collect Ratings and Review Ratings. The RAs will use the Collect Ratings option. They will open their video, put the headphones on, press play, and get to coding. Once the video is over, DARMA asks for a file name and they should name it in the format: Dyad#_Scene_Target (P or T)_Coder initials.csv. The RAs will then save the file locally in a folder with their name. The project manager should then go in and upload the codes to Box once a week.

The Review Ratings option is how you will compare codes. You can load in the video (through the top left “Media” menu option) and the RA codes (through the “Add Annotations” button on the bottom right). Once the codes are in, you can click the “Analyze Ratings” button on the bottom right to see the means, standard deviations, and ICCs for the codes. (An ICC is “a way to decompose variance in ratings to decide how much is systematically related to coders, the stimuli, or other factors, based on different assumptions, in an ANOVA-type framework.”)

Sometimes DARMA freezes ☹. If that happens, search for the Task Manager in the bottom left. In the Task Manager, you can choose to close DARMA (this is like the Force Quit option on Macs).

**Monitoring Data Collection**

In addition to creating coder assignments, the project manager is responsible for monitoring the data collection process. Before data collection, the project manager will set up folders named for each RA. During data collection, the project manager will upload the data from those folders to Box once a week. For assignments longer than one week, the project manager should monitor coder progress on a weekly basis. When an RA still has several videos to code in order to complete an assignment, the project manager should email the RA and provide them with an estimate of how many hours per day they will need to code to complete the assignment. Here is an example email that was sent to a coder a week before an assignment was due:

Hi [name],

This is a friendly reminder that the coding assignment is due by the end of the day Friday, 2/14. As of noon today, you have approximately 12 total hours of coding to complete. To complete the assignment on time will require averaging above 1.5 (~1.7) hours per day of coding. This is a good bit, but also seems doable. As per usual, you can choose to code in whatever time blocks you like (so long as you are able to be attentive!), but I wanted to give you a sense of approximately how much coding time you have left.

Importantly, when coding assignments are longer than one week, the project manager should check the data at least weekly to ensure there are no errors. For instance, we once had a software program error that resulted in 3 weeks of botched codes. Had we been checking the data weekly, at most we would have only lost 1 week of coding to this error.

**Coder Meetings**

To maintain and improve coder reliability, as well as discuss any coding issues that arise while completing an assignment, RAs should meet as a team with the project manager on a weekly basis. Before coder meetings, the project manager should identify which video interactions were the most difficult to code by looking at the ICCs of the group for each scene. The project manager should then code the 5 most difficult scenes so that the group can also look at the reliability codes during the meeting (information regarding how to compare codes is provided above in the section *Software and Saving Data Files*). Meetings will focus on the 5 videos for that week that have the lowest ICCs. RAs will view graphs of their data, watch the identified video interactions, and provide one another with feedback. Coder meetings are interactive and any RA can call ‘stop’ at any time to discuss how a particular moment or set of moments might be coded.

**Data Management**

- In every scene, for each participant, one coder’s time series should be removed (there are 10 codes for each scene so you will end up with 8 for the video)
  - The item-total correlation for each time series (warmth and dominance together) should be computed (i.e. the Pearson correlation between one person’s codes and the average of the group’s). The time series with the lowest item-total correlation should be tossed
- Toss codes for the first 5 seconds of each video to allow time for coders to settle into the task (Sadler et al., 2009)

# **Research Using The Joystick Method**

This section includes a list of papers that have used the joystick method and provides a brief description of each paper.

Sadler, P., Ethier, N., Gunn, G. R., Duong, D., & Woody, E. (2009). Are We on the Same Wavelength? Interpersonal Complementarity as Shared Cyclical Patterns During Interactions. *Journal of Personality and Social Psychology, 97*(6)*,* 1005-1020.

This was the first article written about the joystick method. It describes several aspects of the method (e.g., procedure, correlation and cross-spectral analyses) in great detail. It also provides a base sample to compare future research against (e.g., in terms of the expected range of coherence estimates). The sample included data from 50 unacquainted, mixed-sex, college-student dyads.

Altenstein, D., Krieger, T., & Grosse Holtforth, M. (2013). Interpersonal Microprocesses Predict

Cognitive-Emotional Processing and the Therapeutic Alliance in Psychotherapy for

Depression. *Journal of Counseling Psychology,* *60*(3), 445-452.

The authors examined interpersonal levels and complementarity during a randomly selected session from the middle phase of therapy for 20 depressed individuals. They found that higher client warmth was related to higher reports of the alliance whereas less complementarity occurred at times of greater emotional arousal. They also found evidence that the u-shape of complementarity outlined by Tracey in past research can be found within sessions, although this appears to be hold for affiliation more so than for dominance.

Hopwood, C. J., Harrison, A. L., Amole, M., Girard, J. M., Wright, A. G. C., Thomas, K. M., …

Kashy, D. A. (2018). Properties of the Continuous Assessment of Interpersonal Dynamics

Across Sex, Level of Familiarity, and Interpersonal Conflict. *Assessment*.

This study used the joystick method to understand how sex, familiarity, and conflict affected interpersonal warmth, dominance, and complementarity. They found complementarity effects across all samples. They also found that women were slightly

warmer than men and that unfamiliar dyads were relatively warmer and more submissive compared to familiar dyads.

Klahr, A.M., Thomas, K.M., Hopwood, C.J., Klump, K.L.., & Burt, S.A.. (2013). Evocative

Gene-Environment Correlation in the Mother-Child Relationship: A Twin Study of

Interpersonal Processes. *Development and Psychopathology, 25,* 105–118.

This study demonstrates the utility of using the joystick method to study parent-child dyads and to examine genetic and environmental influences on these relationship processes.

Lizdek, I., Sadler, P., Woody, E., Ethier, N., & Malet, G. (2012). Capturing the Stream of Behavior: A Computer-Joystick Method for Coding Interpersonal Behavior Continuously Over Time. *Social Science Computer Review, 30*(4)*,* 513-521.

This paper describes the joystick method, provides a free version of the software download, outlines the procedure used to collect data, and describes the form the final data take. The authors also discuss several possible applications of the joystick method.

Markey, P. M., Lowmaster, S., & Eichler, W. (2010). A Real-Time Assessment of Interpersonal Complementarity. *Personal Relationships, 17,* 13-25.

This was the first published study using the joystick method after Sadler et al’s (2009) initial publication using the method. In this study, 66 unacquainted female-student dyads interacted, and those who displayed higher warmth complementarity reported liking one another more at the end of the task.

Ross, J. M., Girard, J. M., Wright, A. G. C., Beeney, J. E., Scott, L. N., Hallquist, M. N., …

Pilknois, P. A. (2017). Momentary Patterns of Covariation between Specific Affects and

Interpersonal Behavior: Linking Relationship Science and Personality Assessment.

*Psychological Assessment*, *29*(2), 123–134.

This study used the joystick method to compare two different types of interpersonal

assessment systems – the Specific Affect Coding System and the Continuous Assessment of

Interpersonal Dynamics.

Tracey, T. J. G., Bludworth, J., & Glidden-Tracey, C. E. (2012). Are there Parallel Processes in Psychotherapy Supervision? An Empirical Examination. *Psychotherapy,* *49*(3), 330-343.

In this paper the authors use the joystick method to demonstrate the presence of parallel processes between supervisors/trainees and therapists/clients in each of 17 supervisor-trainee/therapist-client triads.

Thomas, K.M., Hopwood, C.J., Woody, E., Ethier, N., & Sadler, P. (2014). Momentary

Assessment of Interpersonal Process in Psychotherapy. *Journal of Counseling*

*Psychology, 61*(1), 1-14.

This study demonstrates the value of using the joystick method to study psychotherapy process using the Gloria videos as a familiar illustration. The paper describes numerous analytic techniques and their conceptual implications.

**Appendix A: Script for Learning to Use the Joystick**

[**Notes to the Trainer are** **in bold**]

“As the Powerpoint discussed, two important things that we constantly evaluate during social interactions are how friendly people are being, and how dominant they are being, too. I’m eventually going to ask you to use this joystick to evaluate how friendly and dominant you think a person is being when you watch a videotape. But first, I want to show you how this works. We’ll start by getting you to put your hand on the joystick. Good, now move it forward towards the screen. When you do that, it indicates the person is being more dominant; now pull it toward yourself. Good, that indicates the person is being more submissive. Similarly, when you move it to the right (yup do that, too, to get the feel of it), that means the person is being more friendly, and when you move it to the left, that means the person is being more separate. The circle as it moves around tells you where you are on the graph. As you move the joystick around, you’ll notice that you simultaneously indicate how friendly and dominant a person is being. For example, if you think the person is being very friendly and very dominant, where would you put the joystick. **[Say “Good” for correct placement, or provide feedback if incorrect.]** If you thought they were being very friendly but just somewhat dominant, where would you put the joystick? **[Again provide corrective feedback if needed]**. If the person is being slightly separate, and very submissive, where would you put the joystick? **[Provide feedback.]** Good, and if the person is being pretty separate and a bit dominant, where would you put it? Good, you’ve got the hang of this! As a note, the opposite of friendly is separate because the opposite of love is not hate, there is still emotion in hate. The opposite of love is just not caring, so separate could also be labeled as cold or indifferent.

You have probably noticed that the further away from the center that you move the joystick, the more force is applied in the opposite direction. This is to provide some feedback to your hand (tactile feedback) about how far you are pushing the joystick; this force should also help to remind you to move the joystick as you constantly assess how friendly and how dominant the person in the video is being. Now put your fingers just on the top of the joystick and move it around. See how floppy it is? The force feedback is only active when your palm and fingers are fully on the joystick. So when you actually do the real assessments, I will ask you to please keep a reasonably firm grip on the joystick so that the force feedback is constantly active.

Okay, just to make sure you’ve got a good understanding of the entire area where the joystick can be moved, we’re going to practice a bit with a list of words. For this practice session, after each word, I want you to move the joystick all the way to the maximum position where you think the word should belong, just so I can see clearly where you think the best of the 8 positions is for that word. There are 7 words altogether and I’ll write down your answer to each of them; then at the end let you know which ones were incorrect and then we’ll try it again. Okay?”

**[In the table below, write down the person’s answer to each one of the following words under a heading of Trial 1.]**

1. Warm (LM: Friendly)
2. Cold (DE: Separate)
3. Passive (HI: Submissive)
4. Assertive (PA: Dominant)
5. Meek (FG: Separate-Submissive)

PA

HI

LM

BC

NO

JK

FG

DE

1. Attention seeking (NO: Friendly-Dominant)
2. Critical (BC: Separate-Dominant)**ining Procedure Script**

**If all words were correct, then indicate** “Good job. Now you’re ready to give this a try with a video.” **If one or more words were incorrect, then indicate** “Good job, you got XX correct. For **<incorrect word 1>** you said **<incorrect octant>**. **<incorrect word 1>** belongs in **<correct octant>**. Does that make sense?” **[Go through the rest of them that are incorrect in a similar fashion.]** “Okay, now we’re going to go through the 7 words again. Take your time, and place the joystick all the way out, just like last time.” **[Record their answers below and do this until they get all the words correct, and then indicate “Good job. Now you’re ready to give this a try with some videos.”]**

| **Word** | **Trial 1** | **Trial 2** | **Trial 3** |
| --- | --- | --- | --- |
| Warm (LM) |  |  |  |
| Cold (DE) |  |  |  |
| Passive (HI) |  |  |  |
| Assertive (PA) |  |  |  |
| Meek (FG) |  |  |  |
| Attention seeking (NO) |  |  |  |
| Critical (BC) |  |  |  |

# **Appendix B: Script for Introducing Joystick Coding**

You’re soon going to code your first video, in which two people are **[describe task for video to be watched]**.

Okay, let me show you how this works. Watch me as I code the **[person to be coded, e.g., parent]**. It will only be a short demonstration—for about 2 or 3 minutes—but it will probably be useful. Up until now, we haven’t been collecting any data. But for this video we will. You’ll see that as soon as data collection begins, the circle will turn red. That’s what it will be like for this video, and what it will be like when you do the actual assessments after this. **[Trainer codes the joystick video while the trainee watches.]**

What things did you notice about the joystick coding as you were watching? Anything surprise you? Do you have any questions? **[Get them to bring up the following, or raise them yourself with an example or two:**

**- They were able to see why I was moving the joystick one way or another**

**- How fast the joystick was moving**

**- The fluid/flowy nature of joystick coding**

**- Importance of capturing as much variation as possible (so microexpressions are important. If they seem confused, give them examples, like nods, “mm hmm’s”, etc.)]**

Because the scene happens fast, if a person says a dominant thing, you should react right when they say it, you can’t fit it in later. Okay, so now you’re going to give it a try for a 4 minute segment. Now I want you to concentrate on **[person to be coded; this paragraph assumes a female target – change pronouns as needed]**. What I want you to do is think about how dominant and how friendly she is being during the entire time. You get to decide how to make those assessments based on what she is saying and what she is doing. When we did those training words, you moved the joystick always out to the extreme, but now, I would like you to just move it around as her behavior seems to change. Sometimes it might be more extreme (out at the edges like last time) and other times somewhere closer to the middle, or in between. You’ll want to make sure that you are paying attention to various aspects of behavior, such as *what* the person says, *how* she is talking, as well as her body language. So she might do or say something that seems more dominant at one point, and then act a little more submissive a little later, or she might make a lot of eye contact at one point, seeming very friendly, and less eye contact later, seeming less friendly and aloof at another time. Therefore, the joystick should be moving around and you should constantly be asking yourself how friendly is she being now? How dominant is she being now? Make sense?

When doing the actual coding, please wear these headphones. For the training videos, we won’t use them so I can hear the sound coming from the speakers. Do you have any questions? Okay, just do your best.

**Trainee completes joystick assessment with data collected (red dot should be showing on the screen).**

Good. How was that? **[Answer any questions and provide reassurance as needed. Show them where and how to save the codes. Then have trainee code the video again now focusing on the other person and answer questions again. Once they’ve coded both participants, pull up the Review Ratings option and compare their ratings to the reliability codes. Compare the ICCs and give feedback on places they differed from the reliability coder. If their average Average-Score Agreement ICC >= 0.4 on both axes for both participants when compared to the reliability coder move on to the next video].**

This type of comparison with other codes is what we will cover in the weekly meetings. We’ll break down the scene and discuss spots that were harder to code and why. Now you’ll try another 4 minute video and this time you’ll be coding the **[person to be coded]**. Make sure you’re only focusing on that person. Pay attention to what she is doing and saying, and make your assessments based on her behavior and responses during the interaction. People usually find it helpful to rest their arm on the desk during this time. Be careful not to let the joystick slip at any time. Any questions?

# **Appendix C: Joystick Issues That Trainees May Have**

1. **Moving joystick too much**

The joystick is designed so that you pick up on behaviours from second to second, continuously. You are doing a good job moving the joystick around. However, try to not move it as much because the behaviour of the target person you are watching is not changing at such a fast rate. For the coding you need to really focus on this person and watch and joystick their behaviour *as it changes.* Therefore, very rarely would we go from submissive to highly dominant that fast. The ratings are very continuous and flowy in nature.

1. **Not moving the joystick enough**

The joystick is designed so that you pick up on behaviours from second to second, continuously. You are doing a good job moving the joystick around. However, try to move it more than you have been doing because the behaviour of the target person you are watching is changing more than you actually think. For the coding you need to really focus on this person and watch and joystick their behaviour as it changes. Therefore, the behaviour may change from very submissive to dominant or from not very friendly to pretty friendly if the person laughs or makes another friendly gesture. Feel free to use all of the space but of course, pay close attention to what the person is doing and saying. Your ratings should be very continuous and flowy in nature.

1. **Slow reaction time**

The joystick is designed so that you pick up on behaviours from second to second, continuously. You are doing a good job moving the joystick around. However, try to move it as soon as you notice a change in behaviour. Your reaction time needs to be very quick for this. For example, if the person is not very friendly but they all of a sudden laugh, you need to move the joystick to the right as soon as you see this change. For the coding you need to really focus on this person and watch and joystick their behaviour as it changes. Therefore, the behaviour may change from very submissive to dominant or from not very friendly to pretty friendly if the person laughs or makes another friendly gesture. Your ratings should be very continuous and flowy in nature.

1. **Only coding dominant behaviors and ignoring friendliness**

The joystick is designed so that you pick up on behaviours from second to second, continuously. You are doing a good job moving the joystick around, especially picking up in dominant behaviours. However, you need to play close attention to not only dominance, but friendly behaviours as well. Therefore, you need to keep in mind both friendliness and dominance when you are using the joystick. You should always be asking yourself, ‘How friendly is this person being now? How dominant are they being now?’ For example, if the person is not very friendly and not very dominant but they all of a sudden make a joke and laugh, you need to move the joystick to the right (indicating friendliness) and up (indicating dominance) as soon as you see this change. For the coding you need to really focus on this person and watch and joystick their behaviour as it changes. Your ratings should be very continuous and flowy in nature.

1. **Only coding friendly behaviours and ignoring dominance**

The joystick is designed so that you pick up on behaviours from second to second, continuously. You are doing a good job moving the joystick around, especially picking up in friendly behaviours. However, you need to play close attention to not only friendliness, but dominant/submissive behaviours as well. Therefore, you need to keep in mind both friendliness and dominance when you are using the joystick. You should always be asking yourself, ‘How friendly is this person being now? How dominant are they being now?’ For example, if the person is not very friendly and not very dominant but they all of a sudden make a joke and laugh, you need to move the joystick to the right (indicating friendliness) and up (indicating dominance) as soon as you see this change. For the coding you need to really focus on this person and watch and joystick their behaviour as it changes. Your ratings should be very continuous and flowy in nature.

1. **Being too reactive to behaviours**

The joystick is designed so that you pick up on behaviours from second to second, continuously. You are doing a good job moving the joystick around. However, try to not move it as much because the behaviour of the target person you are watching is not changing at such a fast rate. For the coding you need to really focus on this person and watch and joystick their behaviour as it changes. Therefore, very rarely would we go from submissive to highly dominant that fast. The ratings are very continuous and flowy in nature.

1. **Not coding the right person**

You are doing a good job using the joystick. Just to check, which of the two participants were you coding just now? (If says wrong participant, tell them to pay attention to target female only).

1. **Not picking up on something specific (such as nods, smiles, eye contact, etc.)**

The joystick is designed so that you pick up on behaviours from second to second, continuously. You are doing a good job moving the joystick around. However, you need to play close attention to microexpressions as well, such as nods, eye contact, body language, smiles, uh hums, and so on. All of these behaviours will lead you to change your joystick position. For example, if the target person nods, you would move the joystick a little to the right, indicating more friendliness. If the person says a joke and smiles, you would move the joystick upward, to indicate greater dominance, and also to the right, indicating friendliness because they target smiled. For the coding you need to really focus on this person and watch and joystick their behaviour as it changes. Your ratings should be very continuous and flowy in nature.

1. **Confused about what to do**

The joystick is designed so that you pick up on behaviours from second to second, continuously. You are doing a good job moving the joystick around. For the coding you need to really focus on this person and watch and joystick their behaviour as it changes. The ratings are very continuous and flowy in nature. Watch me again so you get a better sense of how this works (Code target example again and talk through coding so that participant knows what is expected of them. Ask if they have any questions and then have the trainee try it again).

# **Appendix D: Lab Cheat Sheet**

**Beginning to Code Steps**

1. Open the laptop & log on
2. See what videos you are assigned to code
   1. “PT” means code the parent first and then the teen
   2. “TP” means code the teen first and then the parent
   3. **Remember to follow the listed scene order**
      1. “Alcohol, Warm Up, Conflict, Marijuana” means code the alcohol video for the participant first and then the warm up for them, then conflict for them, and finally the marijuana video for them
      2. Then switch participants in the video and code in the same scene order
3. When DARMA opens, click “Collect Ratings,” and then click the upper right full screen button
4. Go to the “Media” option in the top menu, click “Open Media”
5. Navigate to the “Coding Project 2019” folder in the left side of the window
6. Open the “Videos” folder
7. Open the **correct scene folder** listed on the spreadsheet
8. Open the **correct dyad video** listed on the spreadsheet
9. Click the “Begin Ratings” button
10. **Focus only on the person you are supposed to**

**Finished Coding Steps**

1. Once you are done coding, save your file in **your named folder** in “Coding Project 2019”
   1. File name format: Dyad#_ Scene_Target (P or T)_Coder initials.csv
      1. Ex: 04_WarmUp_T_JV.csv
2. Replace the PT or TP letters in the spreadsheet with X’s for who you coded
3. Write down any comments you have about the video in the comments column

**Breaks**

- Try to get through a whole dyad and then take a break
- You should be watching all the videos for 1 participant and then switching
  - If you can’t do all of the dyad videos without taking a break, try to get through a whole participant in a dyad (all 4 videos)
- Do not stop a video in the middle, always code the whole video in 1 sitting
  - DARMA does not record the coding until the end of the video

**Reminders**

- Move the joystick!
- Go with whatever your instinct is, don’t over think it
- Code all dyad scenes in one sitting
  - If you can’t do that code all of 1 participant in a dyad in one sitting
- Always code all the way through a video
- Take breaks after videos if your attention is going!

**Possible Issues & Their Solutions**

- If DARMA freezes
  - Search for the Task Manager in the bottom left search bar
  - In the Task Manager, you can choose to close DARMA (this is like the Force Quit option on Macs)
- If a video won’t resize properly
  - Quit DARMA and reopen the video
- If there is no sound in the whole video
  - Make a note in the comments section for that dyad
  - Skip that video
