# Supplementary material for: Variance Decomposition of the Continuous Assessment of Interpersonal Dynamics (CAID) system: Assessing sources of influence and reliability of observations of parent-teen interactions
Source: PLoS One. 2023 Oct 18;18(10):e0292304. doi: 10.1371/journal.pone.0292304 (PMC10584132; doi:10.1371/journal.pone.0292304)
Supplement: S1 Table — (DOCX) [file pone.0292304.s003.docx]

Table 6. Variance Parameters

|  | **Indices of Individual Behavior** | | | | | **Indices of Dyadic Complementarity** | | | |
| --- | --- | --- | --- | --- | --- | --- | --- | --- | --- |
|  | Mean | | Variability | | Shape | Mean-Level | | Moment-to-Moment | |
| *Source of Variance* | *Warm* | *Dom* | *Warm* | *Dom* | *Warm-Dom* | *Warm* | *Dom* | *Warm* | *Dom* |
| σ^2^ Person | 14.9%  3178.96^***^ | 20.6%  4874.13^***^ | 3.1%  84.60^***^ | 1.4%  45.88^*^ | 1.5%  0.001 | -- | -- | -- | -- |
| σ^2^ Dyad | 19.9%  4241.93^***^ | 0.0%  0.0 | 5.0%  136.44^*^ | 6.8%  230.81^***^ | 4.0%  0.004^*^ | 30.2%  2770.51^***^ | 9.9%  907.70^***^ | 3.6%  0.001 | 27.5%  0.02^***^ |
| σ^2^ Situation | 6.8%  1450.03^**^ | 0.0%  0.001 | 5.3%  143.48^***^ | 3.2%  107.50^**^ | 6.5%  0.01^*^ | 0.3%  27.84 | 3.4%  312.20^***^ | 0.2%  <0.001 | 6.6%  0.003^***^ |
| σ^2^ Rater | 10.0%  2134.62^***^ | 0.0%  0.23 | 23.5%  636.29^***^ | 41.3%  1403.54^***^ | 6.7%  0.01^*^ | 4.6%  419.74^***^ | 14.9%  1372.60^***^ | 5.6%  0.002^***^ | 22.6%  0.01^***^ |
| σ^2^ Person*Situation | 3.6%  770.08^***^ | 8.2%  1937.58^***^ | 2.3%  63.39^***^ | 0.4%  13.33 | 6.3%  0.01^***^ | -- | -- | -- | -- |
| σ^2^ Dyad*Situation | 5.5%  1180.41^***^ | 0.0%  0.0 | 5.2%  141.78^***^ | 5.6%  188.92^***^ | 3.1%  0.003^*^ | 6.4%  582.05^***^ | 7.1%  652.60^***^ | 13.6%  0.004^***^ | 15.5%  0.01^***^ |
| σ^2^ Rater*Situation | 2.2%  472.90^***^ | 0.2%  36.70^***^ | 3.6%  96.65^***^ | 1.0%  33.93^***^ | 2.7%  0.003^***^ | 0.4%  34.73 | 1.9%  177.90^***^ | 0.0%  <0.001 | 0.8%  <0.001^**^ |
| σ^2^ Person*Rater | 7.6%  1618.65^***^ | 9.8%  2314.03^***^ | 5.3%  143.14^***^ | 9.8%  331.25^***^ | 15.4%  0.02^***^ | -- | -- | -- | -- |
| σ^2^ Dyad*Rater | 14.3%  3059.76^***^ | 0.0%  4.84 | 22.6%  612.02^***^ | 14.2%  482.03^***^ | 11.1%  0.01^***^ | 25.7%  2354.59^***^ | 35.8%  3288.10^***^ | 8.2%  0.003^***^ | 8.8%  0.01^***^ |
| σ^2^ Kinsperson sex | 2.2%  463.25 | 45.5%  10740.51^***^ | 0.0%  <0.001 | 0.0%  <0.001 | 5.5%  0.01^*^ | -- | -- | -- | -- |
| σ^2^ Kinsperson*Situation | 0.0%  4.84 | 2.8%  656.70^***^ | 0.0%  0.0 | 0.1%  2.95 | 0.4%  <.001 | -- | -- | -- | -- |
| σ^2^ Kinsperson*Rater | 1.1%  242.85^**^ | 6.8%  1607.73^***^ | 0.5%  12.36 | 3.1%  105.25^***^ | 2.3%  0.002^***^ | -- | -- | -- | -- |
| σ^2^ Residual Error | 11.9%  2530.51 | 6.2%  1456.43 | 23.5%  636.49 | 13.3%  450.73 | 34.4%  0.03 | 32.5%  2975.84 | 27.0%  2480.81 | 68.8%  0.02 | 18.2%  0.01 |

*Note:* Analyses followed Cranford and colleagues’ [50] analysis of variance model for estimating sources of variance. Each column represents the respective CAID parameter, whereas each row represents the source of influence on CAID parameter variance (top row = variance proportions, bottom row = variance parameter). Possible sources of variance included persons (n = 122), Dyads (n = 61), situations (n = 4), rater (n = 10), Kinsperson sex (n = 2), and their relevant interactions. Double dash marks (--) indicate not applicable (as Kinsperson variables are not involved in computing dyadic complementarity variables). Variation proportions within each column sum to 100%, within rounding error. Significance for random effects (i.e., variance parameters) were determined with Likelihood-Ratio tests within each parameter using lmerTest package in R (* p ≤ .05, ** p ≤ .01, *** p ≤ .001).
